# The GSK3 kinase and LZTR1 protein regulate the stability of Ras family proteins and the proliferation of pancreatic cancer cells

**DOI:** 10.1016/j.neo.2022.01.002

**Published:** 2022-01-31

**Authors:** Chitra Palanivel, Neha Chaudhary, Parthasarathy Seshacharyulu, Jesse L. Cox, Ying Yan, Surinder K. Batra, Michel M. Ouellette

**Affiliations:** a Department of Internal Medicine, University of Nebraska Medical Center, Omaha, NE 68198, USA.; b Department of Biochemistry and Molecular Biology, University of Nebraska Medical Center, Omaha, NE 68198, USA.; c Department of Pathology and Microbiology, University of Nebraska Medical Center, Omaha, NE 68198, USA.; d Department of Radiation Oncology, University of Nebraska Medical Center, Omaha, NE 68198, USA.

**Keywords:** Pancreatic cancer, RAS, LZTR1, GSK3, Protein stability

## Abstract

Ras family proteins are membrane-bound GTPases that control proliferation, survival, and motility. Many forms of cancers are driven by the acquisition of somatic mutations in a *RAS* gene. In pancreatic cancer (PC), more than 90% of tumors carry an activating mutation in *KRAS*. Mutations in components of the Ras signaling pathway can also be the cause of RASopathies, a group of developmental disorders. In a subset of RASopathies, the causal mutations are in the LZTR1 protein, a substrate adaptor for E3 ubiquitin ligases that promote the degradation of Ras proteins. Here, we show that the function of LZTR1 is regulated by the glycogen synthase kinase 3 (GSK3). In PC cells, inhibiting or silencing GSK3 led to a decline in the level of Ras proteins, including both wild type Ras proteins and the oncogenic Kras protein. This decline was accompanied by a 3-fold decrease in the half-life of Ras proteins and was blocked by the inhibition of the proteasome or the knockdown of LZTR1. Irrespective of the mutational status of *KRAS*, the decline in Ras proteins was observed and accompanied by a loss of cell proliferation. This loss of proliferation was blocked by the knockdown of LZTR1 and could be recapitulated by the silencing of either KRAS or GSK3. These results reveal a novel GSK3-regulated LZTR1-dependent mechanism that controls the stability of Ras proteins and proliferation of PC cells. The significance of this novel pathway to Ras signaling and its contribution to the therapeutic properties of GSK3 inhibitors are both discussed.

## Introduction

Ras proteins are membrane-bound GTPases implicated in the regulation of cell motility, proliferation, and survival [1,2]. Ras proteins exhibit high-affinity binding to GDP and GTP and act as binary switches. Ras proteins cycle between an active GTP-bound state and inactive GDP-bound form, and the ratio between these forms is regulated by GEFs (guanine exchange factors) and GAPs (GTPase-activating proteins), which are, in turn, regulated by upstream growth factor receptors. GEFs activate Ras proteins by promoting the release of GDP and loading of GTP, whereas GAPs deactivate them by stimulating their intrinsic GTPase activity. In their active GTP-bound state, Ras proteins interact with and activate their downstream effectors, many of which involved in promoting proliferation and survival (MAPK, PI3K, Rac1 pathways). Defects in Ras signaling have been associated with cancers and can also be the source of developmental disorders, termed RASopathies.

More than 30% of all human tumors carry an oncogenic mutation in a *RAS* gene, most commonly the *HRAS, KRAS*, or *NRAS* gene. Pancreatic cancer (PC) is the prototypical Ras-driven cancer. Oncogenic *KRAS* mutations are the earliest and most commonly detected genetic alterations in PC 3., 4., 5., 6., 7.. Close to 95% of PC tumors carry an activating mutation in the *KRAS* gene, almost always at codon 12. These mutations impair the GTPase activity of the Kras protein and its interaction with GAPs, which leaves Kras constitutively activated along with its downstream effectors [8,9]. In mice, the pancreas-specific expression of oncogenic *KRAS* drives the formation of PanIN precursor lesions and cooperates with the loss of p53 (encoded by the mouse *Trp53* gene) to give rise to PC 10., 11., 12.. In these animals, the tumors that form are addicted to the *KRAS* oncogene, to the extent that its subsequent repression results in cell death and tumor regression 13., 14., 15., 16.. In a recent report, more than 50% of human PC cell lines were addicted to oncogenic *KRAS*, especially those exhibiting a ductal epithelial phenotype [17]. In *KRAS*-addicted lines, but not in normal cells or *KRAS*-independent cancer cells, this repression leads to the induction of apoptosis 16., 17., 18.. This important role of oncogenic Ras proteins in tumor maintenance has made them prime targets for the development of novel cancer therapies [8,9].

Malignancies are not the only maladies associated with Ras mutations. RASopathies are a group of rare developmental disorders caused by mutations in components of the Ras-MAPK pathway [19,20]. Some of these mutations elevate Ras signaling in tissues during development, which leads to malformations and developmental defects [19,20]. In Noonan syndrome, the causal mutation is frequently found in the *LZTR1* gene 21., 22., 23., 24., 25., 26.. The LZTR1 protein is the substrate adaptor for an E3 ubiquitin ligase complex that targets Ras family proteins for proteasomal degradation, including the Kras, Hras, Nras, Mras, Rit1, and Rin proteins [21,22,24]. LZTR1 has a Kelch domain that binds Ras family proteins and a pair of BTB-BACK domains with which it interacts with the Cul3 protein. The resulting LZTR1-Cul3-Rbx1 trimer (BCR^LZTR1^ ligase) catalyzes the K48-linked polyubiquitination of Ras proteins and regulates their stability [21,22,24]. How the function of these LZTR1-directed E3 ligase complexes is regulated under normal and pathological conditions is not well understood.

Glycogen synthase kinase 3 (GSK3) is a highly-conserved ubiquitously expressed serine/threonine protein kinase. In humans, the enzyme is comprised of two related isoforms produced by separate genes, GSK3α and GSK3β 27., 28., 29.. The kinase is found in virtually all subcellular compartments, sometimes in association with other proteins that carry GSK3β-interacting domains (GID) [28,30., 31., 32.]. GSK3 has a preference for primed substrates, which have already been phosphorylated by another kinase [28,29]. GSK3 phosphorylates serine/threonine residues when located 4 amino acids upstream of an already phosphorylated serine or threonine (i.e. (S/T)XXX(S/T) sites, in which the underlined S/T must first be phosphorylated). Unlike other kinases, GSK3 is constitutively active under resting conditions and is instead regulated through its inhibition, by for example the AKT kinase [27]. In PC specimens, GSK3β is reportedly overexpressed 33., 34., 35. and in mouse models of *KRAS*-driven PC [10], GSK3β is required for acinar-to-ductal metaplasia (ADM), an early manifestation of oncogenic *KRAS* signaling [36]. In the GSK3β-deficient animals, the formation of PanIN precursor lesions was delayed and disease progression was blunted. Further, in animals with pre-established pancreatic tumors, GSK3 inhibitors could block tumor growth [37] and sensitize tumor cells to DNA damaging agents [35,38,39]. In a recent screen for drugs that can selectively kill Ras-addicted cancer cells, inhibitors of GSK3 were identified as potent candidates [40]. In a panel of cancer cell lines, GSK3 inhibition induced apoptosis in the Ras-dependent cell lines, but not in Ras-independent lines [40]. This induction of apoptosis was accompanied by the accumulation of c-Myc and β-catenin proteins and reportedly took place with little to no change in the level of Ras proteins [40].

In PC cell lines, we investigated the effects of GSK3 inhibition on the Ras signaling pathway. In PC cells, GSK3 deficiency led to a degradation of Ras family proteins, including both wild-type Ras proteins and the oncogenic Kras protein. This loss of Ras proteins was dependent on the expression of LZTR1 and was accompanied by an inhibition of proliferation. This inhibition of proliferation was blocked by the knockdown of LZTR1 and could be recapitulated by the silencing of either KRAS or GSK3. These findings reveal a novel GSK3-regulated LZTR1-mediated mechanism that controls the stability of Ras family proteins and the proliferation of PC cells. The potential significance of this new mechanism in Ras signaling and its potential contribution to the therapeutic properties of GSK3 inhibitors are discussed.

## Materials and methods

### Materials

Fetal bovine serum (FBS) was from Atlas Biologicals (Fort Collins, CO). Gentamycin, Penicillin/Streptomycin, Dulbecco's modified Eagle's medium (DMEM), and recombinant human EGF were purchased from ThermoFisher Scientifics (Waltham, MA). Medium M3 (cat# M3: BaseF) was from InCell Corp. (San Antonio, TX). Insulin Aspart (NovoLog®; 100 U/ml) was purchased from the UNMC pharmacy. Cycloheximide and the mammalian proteases inhibitor cocktail were from Sigma-Aldrich (Saint-Louis, MO). All other chemicals were from purchased from Fisher Scientific (Pittsburgh, PA, USA). CHIR98014 (catalogue # S2745) was obtained from Selleck Chemicals (Houston, TX, USA). MG132 (cat# BML-PI102-0025) was purchased from Enzo Life Sciences, Inc. (Farmingdale, NY, USA), dissolved in DMSO, and stored at -80°C.

### Cell lines

The AsPC1, HPAF/CD18, L3.6pl, and BxPC3 cells used in the experiments were authenticated by STR profiling performed by Genetica, LabCorp (Burlington, NC). The first three lines were cultivated in DMEM media supplemented with 10% FBS and 50 µg/ml gentamycin. BxPC3 cells were cultivated in RPMI media, also supplemented with 10% FBS and 50 µg/ml gentamycin. hTERT-HPNE cells (referred therein as HPNE cells) are a line of human pancreatic ductal cells previously immortalized by us using the catalytic subunit of telomerase [41,42]. HPNE cells were cultivated in medium “D”, as described before [42]. All cell lines were cultivated at 37°C in a humidified atmosphere containing 5% CO_2_.

### siRNA knockdowns

Cells were reverse transfected with siRNA using DharmaFECT 1 (cat# T-2001) according to the manufacturer's instructions (Dharmacon, Lafayette, CO). Two days later, cells were examined for expression of the knocked-down targets (GSK3α, GSK3β, LZTR1, and Kras proteins) and for differences in Ras protein level or stability. ON-TARGETplus siRNA were purchased from Dharmacon (Lafayette, CO), including the non-targeting control pool (cat# D-001810-10) and SMARTpools against *GSK3A* (cat# L-003009-00), *GSK3B* (cat# L-003010-00), *LZTR1* (cat# L-012318-00), or *KRAS* (cat# L-005069-00).

### Western blot analysis

With a rubber policeman, adherent cells were released into the medium, after which cells were recovered by centrifugation (300 g x 5 min), lysed in Laemmli buffer (200 µl per 35 mm dish), and stored at -20°C. Equal volume of each samples (20-35 µl) were analyzed by Western blot analyses, as previously described [43]. When probing for proteins of identical sizes (e.g. pERK(T202/Y204) and total ERK), two approaches were alternatively used. In the first approach, the same samples were serially loaded on multiple gels to produce replicate membranes that were subsequently probed separately with the different antibodies. In the second approach, a single membrane was produced, probed with the first antibody, stripped, and subsequently re-probed with the second antibody. Ponceau S staining was used to confirm equal loading and transfer. GAPDH and/or β-actin were also used as internal controls, whose levels were not expected to change during the treatments. Ras family proteins were detected using a pan Ras antibody that recognizes Hras, Kras, and Nras (RAS10 antibody) [44]. An antibody that binds selectively to the G12D mutants of Ras proteins (Ras^G12D^) was also used (cat# 26036; NewEast Biosciences, King of Prussia, PA). Antibodies against GAPDH (cat# sc-47724), β-actin (cat# sc-1616), LZTR1 (cat# sc-390166), ERK (cat# sc-154-G), p-ERK(T202/Y204)(cat# sc-7383) were from Santa Cruz Biotechnology (used at 1:200 dilution). Rabbit monoclonal antibodies against cleaved caspase 3 (clone 5A1E), AKT (clone C67E7), p-AKT(T308) (clone D25E6), p-AKT(S473) (clone D9E), GSK3α (clone D80E6), p-GSK3α(S21) (clone D1G2), GSK3β (clone D5C5Z), p-GSK3β(S9) (clone D85E12), cMyc/N-Myc (clone D3N8F), and p-cMyc(T58) (clone E4Z2K), GS (clone 15B1), and p-GS(S641) (clone D4H1B) were from Cell Signaling Technology (mostly used at 1:1000 dilution). Secondary antibodies used were horseradish peroxidase-conjugated goat antibodies against mouse or rabbit IgG (Jackson ImmunoResearch). Size markers used were the Precision Plus Protein™ Dual Color Standards (cat# 1610374) from Bio-Rad (Hercules, CA).

### Measuring Ras protein stability

In duplicates, AsPC1 cells were reversed transfected with the different siRNA SMARTpools (NT, GSK3α, GSK3β, or GSK3α+β). Two days later, cycloheximide (CHX; 50 µg/ml) was added to block protein synthesis and samples were collected before (t = 0) and at different times after CHX. Levels of Ras (pan Ras) and Actin (β-Actin) proteins were quantified by Western blot and signals were quantified using the ImageJ program. Relative amounts of Ras proteins (normalized to 1 for t = 0) were plotted as a function of time after CHX addition (Fig. 2D), and the data was fitted by non-linear regression to an exponential decay curve to allow calculation of Ras Proteins half-lives under each condition (Fig. 2E). Ras protein half-lives were estimated as the mean ± S.D. of two independent experiments done in parallel in AsPC1 cells.

### Quantitation of the KRAS mRNA by real-time RT-PCR

RNA were isolated from independently treated triplicates with TRIzol (ThermoFisher Scientifics). Isolated RNA were reversed transcribed (1 µg RNA/reaction) using the iScript™ Reverse Transcription Supermix according to the manufacturer's instructions (Bio-Rad). Quantitation of the abundance of KRAS and GAPDH transcripts was done by real-time PCR. TaqMan Gene Expression Assays with FAM-conjugated MGB (minor groove binder) probes were used for the quantification of KRAS (cat# Hs00364282_m1) and GAPDH (cat# Hs99999905_m1) transcripts. These MGB probes incorporate a 5′-FAM reporter dye and a 3′ non-fluorescent quencher (NFQ). Standard curves were produced to allow for the precise calculation of the abundance of each transcript. PCR was performed in a Light cycler 480 II PCR System (PCR System, Roche Applied Science).

### Measuring cell proliferation

In 6-well plates, each cell line was seeded in duplicates at 2.5-5.0 × 10^4^ cells/well, depending on the cell line. The next day, cells were given fresh medium containing different concentrations of CHIR98014 (0 to 10 µM). On day 0 and after 1, 2, and 3 days of each treatment, duplicate dishes were harvested and immediately fixed and stained with crystal violet. Under the microscope, cells were counted in 5 random fields/well to produce an average cell count for each well. For each concentration of CHIR98014 (0, 0.5, 1, 2, 5, and 10 µM), cell numbers were plotted for each day of treatment (0, 1, 2, and 3 days) as the mean ± S.D. of two or three independent wells (*n* = 2 or 3), depending on the experiment. Proliferation rates were estimated based on the numbers of cells counted after 3 days of treatment. Proliferation rates were expressed in population doublings per day (PD/day) and were plotted as a function of the concentration of CHIR98014 to produce a dose-response curve. For each curve, an EC_50_ value was calculated by non-linear regression and fitted to a four parameter logistic curve by SigmaPlot v. 11.

### CHIR99021 treatment of mice implanted with PC tumor cells

All animal experiments were reviewed and approved by the Institutional Animal Care and Use Committee of the University of Nebraska Medical Center. Xenograft study was performed as described previously [45,46]. Briefly, AsPC1 cells (1 × 10^6^ viable cells in 50 µL PBS) were subcutaneously implanted in the right flank of ten 6-8 weeks old athymic nude mice (NU/J; in house breeding). Two weeks later, tumor volumes were measured with a digital caliper and mice were randomized into two groups (*n =* 5 per group) receiving either half the maximum tolerated dose of CHIR99021 (37.5 mg/kg twice/day by oral gavage; [47]) or vehicle (PBS). Every 3-4 days, mice were weighted and tumor volumes were measured with a digital caliper. Tumor volumes were calculated using the formula for an hemiellipsoid (volume = 0.5236 × length × width × height), as this form best approximated the tumors’ shapes. Mice were treated 5 days/week for 16 days, after which the animals were sacrificed. At the end of the experiment, tumors were harvested, weighted, and cut in two halves. The first half of each tumor was pulverized in liquid nitrogen and subsequently lysed in Laemmli buffer (4 ml/g of tissues) with the help of a loose and then tight fitting Dounce pestles. After sonication, samples were cleared by centrifugation (12,000 g for 5 minutes) and the supernatants were collected, heated at 95°C for 2 minutes, and stored at -20°C. An equal volume of each extract was analyzed by Western blot. The second half of each tumor was formalin-fixed, paraffin embedded, and set aside for immunohistochemical (IHC) analysis.

### Immunohistochemical analysis

IHC analysis of tumor specimens was performed as we have done previously [48]. Antibodies used included a rabbit polyclonal antibody against cleaved caspase 3 (Cell Signaling Technology # 9661; used at a 1:200 dilution) and a mouse monoclonal antibody against Ki-67 (Cell Signaling Technology # 9449; used at a 1: 400 dilution). Numbers of positive cells per high power field were quantified by one of us, board certified pathologist Jesse L. Cox.

## Results

### GSK3 inhibition reduces the level of Ras family proteins in PC cells

The GSK3 isoforms had been reported to be essential to the viability of oncogenic *KRAS*-addicted cancer cells, but to be dispensable to Ras-independent cancer cells [40]. To follow-up on this report by another group, we sought to examine the effects of GSK3 inhibition on the Ras signaling pathway itself. Initial experiments were performed in pancreatic cancer (PC) cell lines AsPC1 and HPAF/CD18, both carrying an oncogenic mutation in the *KRAS* gene [49,50].

**Figure 1:**
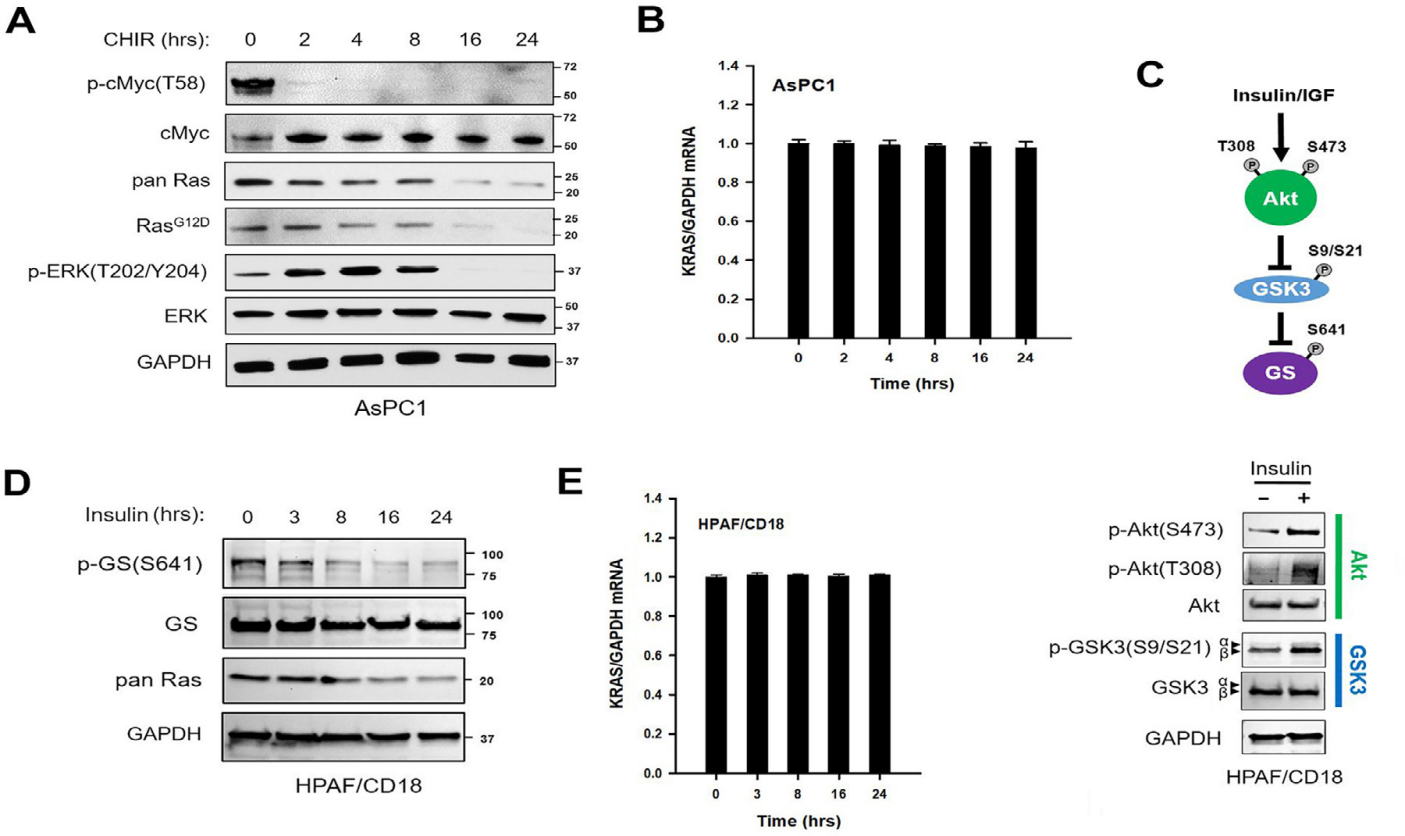
GSK3 inhibition reduces the level of Ras family proteins. (A) GSK3 inhibition reduces Ras signaling and the level of Ras proteins. AsPC1 cells were harvested at the indicated time points after the addition of CHIR98014 (10 µM). Samples were Western blotted with the indicated antibodies, including Ras family proteins (pan Ras) and their G12D mutant proteins (Ras^G12D^). Phosphorylated and total cMyc proteins were used as surrogate markers of GSK3 kinase activity. Positions of Bio-Rad dual color standards are shown in KDa. The experiment was done twice with the same outcome. (B) Abundance of the *KRAS* mRNA is unchanged after GSK3 inhibition. AsPC1 cells were harvested at the indicated time points after CHIR98014 (10 µM). Real-time RT-PCR was used to quantify the abundance of KRAS and GAPDH mRNA. KRAS/GAPDH mRNA ratio is shown as the mean ± S.D. of triplicate samples (*n =* 3). (C) Insulin induces the phosphorylation and inhibition of GSK3. Top panel: Insulin/IGF signaling has been shown to promote the T308- and S473-phosphorylation and activation of the Akt kinase. Akt can then phosphorylate GSK3α (at S21) and GSK3β (at S9), thereby inhibiting the two kinases. This inhibition of GSK3 allows for the activation of glycogen synthase, which otherwise is kept inhibited by the phosphorylation of its S641 residue by GSK3. Bottom panel: HPAF/CD18 cells were exposed to Insulin Aspart (0.04 U/ml). Three hours later, cells were examined for changes in Akt and GSK3 phosphorylation. The experiments was repeated 3 times with the same outcome. (D) Insulin reduces the level of Ras family proteins. The levels of Ras proteins (pan Ras) and S641-phosphorylated glycogen synthase (p-GS) were monitored in HPAF/CD18 cells after the addition of Insulin Aspart (0.04 U/ml). The experiment was repeated twice with the same results. (E) *KRAS* mRNA is unchanged after the addition of Insulin Aspart (0.04 U/ml). AsPC1 cells were harvested at the indicated time points after insulin. KRAS/GAPDH mRNA ratio is shown as the mean ± S.D of triplicate samples (*n =* 3).

In a first series of experiments, PC cells were exposed to CHIR98014, a GSK3 inhibitor that selectively blocks the two isoforms of GSK3 [51]. In pilot studies, the drug inhibited colony formation with EC_50_ values in the range of 1-4 µM (Fig. S1). In a first series of experiments, we treated AsPC1 cells and HPAF/CD18 cells with 10 µM CHIR98014 and examined the cells for changes in levels of Ras proteins and markers of Ras signaling. To quantify Ras proteins, we have used two antibodies: a pan Ras antibody against the Hras, Kras, and Nras proteins (pan Ras antibody)(44) and a second antibody that binds selectively to their G12D mutants (Ras^G12D^ antibody), employed here to detect the oncogenic Kras^G12D^ protein. Used as surrogate markers of GSK3 kinase activity, the T58-phosphorylation of cMyc and level of cMyc protein were also monitored. The T58-phosphorylation of cMyc by GSK3 promotes its proteolytic degradation [52]. As expected, CHIR98014 led to a rapid loss of cMyc T58-phosphorylation and concomitant increase in total cMyc protein, all of which indicative of GSK3 inhibition. Also starting after 2 hours of exposure, a slow decline in Ras proteins (pan Ras) and oncogenic Kras protein (Ras^G12D^) was observed, with both markers reaching their lowest levels by 16 hours (Figs. 1A, S2). Downstream of Ras proteins [53], the ERK kinases were initially activated by the GSK3 inhibitor, between 2 and 8 hours of exposure, but as Ras proteins continued to decline, this activation was eventually followed by a complete inhibition of the ERK kinases by 16 hours of exposure (Figs. 1A, S2). To determine if the declining levels of Ras proteins were driven by changes at the mRNA level, we have measured the *KRAS* mRNA by quantitative real-time RT-PCR. No changes in the abundance of the *KRAS* mRNA were observed in response to the GSK3 inhibitor (Fig. 1B).

GSK3 is typically active in resting cells, but can be inhibited by insulin signaling to promote glycogen synthesis [27](Fig. 1C; top panel). Insulin signaling stimulates the PI3K-Akt pathway, which results in the Akt-mediated phosphorylation of GSK3α (at S21) and GSK3β (at S9). This phosphorylation inhibits GSK3 and allows for the stimulation of glycogen synthase (GS), whose activity is otherwise inhibited by the phosphorylation of its S641 residue by GSK3. In HPAF/CD18 cells, exposure to insulin activated the PI3K/Akt/GSK/GS cascade (Fig. 1C; bottom panel). Three hours after insulin, Akt was activated (S308- and S473-phosphorylation) and GSK3 was inhibited, as indicated by the increase in S9/S21-phosphorylated GSK3 and reduced phosphorylation of GS. Next, we assessed the effects of insulin of the level of Ras proteins. HPAF/CD18 cells were exposed to 0.04 U/ml of Insulin Aspart, after which Ras family proteins were monitored. Employed as a surrogate marker of GSK3 kinase activity, the level of S641-phosphorylated GS was also monitored. Insulin led to a time-dependent decline in the level of both S641-phosphorylated GS and Ras proteins (Fig. 1D). By 16 hours of exposure, a parallel decrease in both markers was clearly observed. We also have quantified the KRAS mRNA by real-time RT-PCR. Again, no changes in the abundance of the KRAS mRNA were observed in response to GSK3 inhibition, this time elicited by insulin (Fig. 1E). Collectively, the results suggested that the activity of GSK3 was regulating the abundance of Ras proteins in PC cells, at the level of either Ras protein stability and/or mRNA translation.

### Silencing of GSK3 reduces the level and stability of Ras family proteins in PC cells

**Figure 2:**
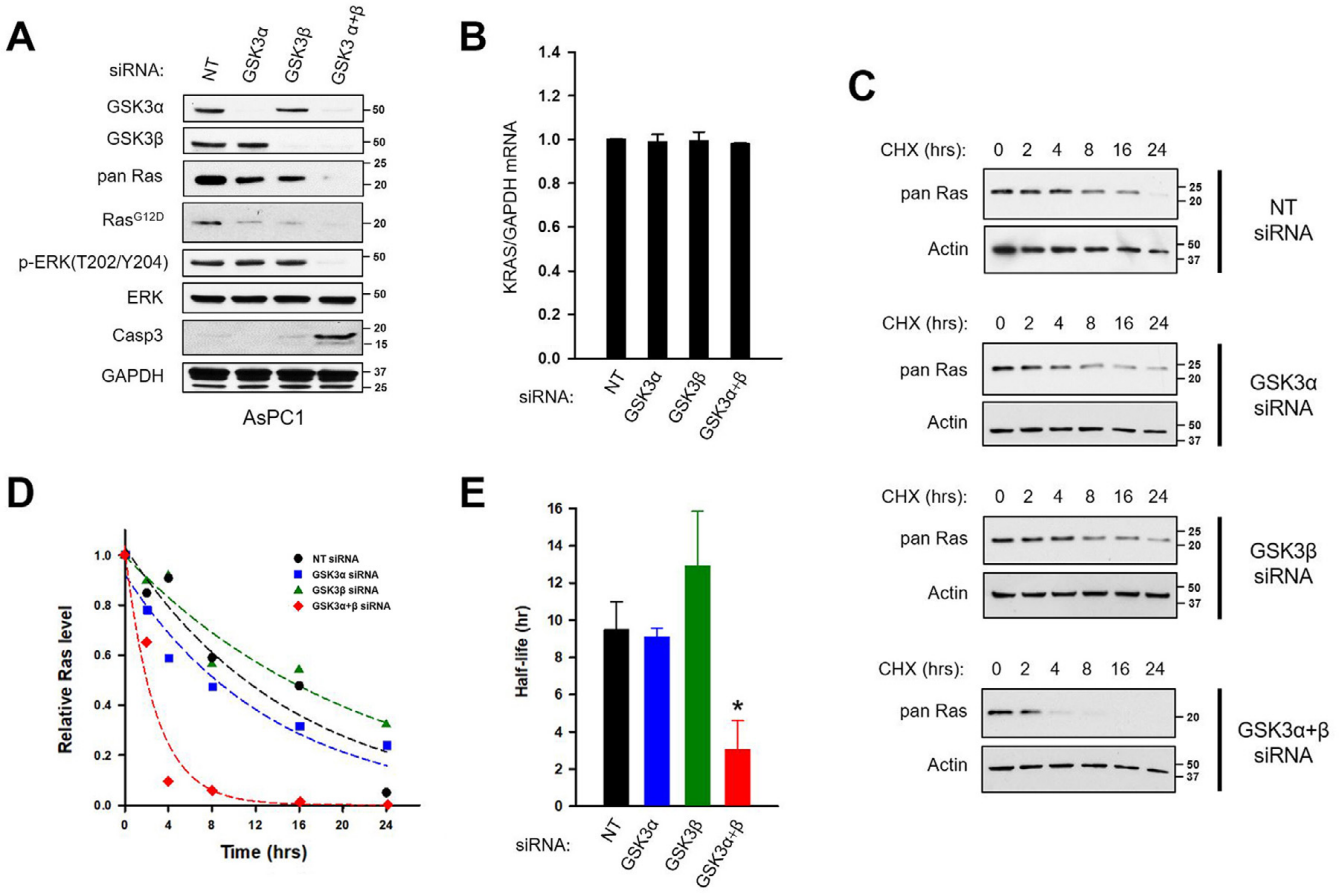
GSK3 silencing reduces the stability of Ras family proteins. AsPC1 cells were transfected with a non-targeting (NT) siRNA or with siRNA against GSK3α alone, GSK3β alone, or both kinases. Two days later, cells were analyzed by Western blotting (panel A) or real RT-PCR (panel B) or else were exposed to cycloheximide (panels C-E). (A) The silencing of GSK3 reduces the level of Ras proteins. Two days post-transfection, cells were analyzed by Western blotting for the presence of the indicated proteins. Positions of Bio-Rad dual color standards are shown in KDa. The experiment was done twice with same outcome. (B) The abundance of the KRAS mRNA remains unchanged after the silencing of GSK3. Two days post-transfection, RNA samples were isolated and subjected to real-time RT-PCR quantification of KRAS and GAPDH transcripts. KRAS/GAPDH mRNA ratio is shown as the mean ± S.D of triplicate samples (*n =* 3). (C) GSK3α and GSK3β co-regulate the stability of Ras family proteins. Two days post-transfection, duplicate wells cells were exposed to cycloheximide (CHX) and the level of Ras proteins was monitored over time (Pan Ras). Actin, a protein known to have a much longer half-life, was used as an internal control. (D) Line graph shows the level of Ras proteins as a function of time after CHX addition. Levels are shown for cells transfected with the non-targeting siRNA (black circles) or with siRNA against GSK3α (blue squares), GSK3β (green triangles), or both kinases (red diamonds). Dotted lines are non-linear regressions of each data set to an exponential decay equation. (E) Bar graph shows the calculated half-lives of Ras proteins under the four conditions. Mean ± S.D. of two independent experiments.

To confirm the involvement of GSK3 in the regulation of Ras protein levels, AsPC1 cells were transfected with siRNA against GSK3α alone, GSK3β alone, or both kinases (Fig. 2A). Cells transfected with a non-targeting siRNA were used as controls. Two days later, cells were analyzed for differences in the level of Ras proteins (Fig. 2A) and KRAS mRNA (Fig. 2B) and were also used for determination of the half-life of Ras proteins (Fig. 2C-E). Two days post-transfection, the depletion of GSK3α and GSK3β was almost complete (Fig. 2A). In cells depleted of both GSK3 isoforms, Ras family proteins were greatly reduced to the limit of detection (pan Ras), including the oncogenic Kras^G12D^ protein (Ras^G12D^). Downstream of Ras, the phosphorylation and activation of ERK correlated with the level of Ras proteins and was undetectable after the silencing of both GSK3 isoforms. In *KRAS*-addicted cancer cells, interrupting Ras-ERK signaling can induce apoptosis [14,17,18]. In line with this expected response, the loss of Ras proteins was accompanied by the induction of apoptosis (Fig. 2A; cleaved Casp3). Depleting just one isoform of GSK3, either GSK3α or GSK3β, led to more modest decreases in Ras proteins (Pan Ras, Ras^G12D^) and did not suffice to reduce pERK or induce apoptosis. We also have measured the abundance of the *KRAS* mRNA by real-time RT-PCR (Fig. 2B). In cells transfected with the GSK3 siRNA, the abundance of the *KRAS* mRNA was unchanged (Fig. 2B).

Certain members of the Ras family are regulated at the level of protein stability [21,22,24]. To investigate this possibility, we used the cycloheximide chase assay [54] to measure the half-life of Ras family proteins. Two days post-transfection, AsPC1 cells transfected with the different siRNA (NT, GSK3α, GSK3β, and GSK3α+β) were exposed to cycloheximide (50 µg/ml) to block protein synthesis, after which Ras proteins was monitored over time (Fig. 2C-D). In cells transfected with the NT siRNA, Ras proteins were determined to have a relatively short half-life (9.5 ± 1.5 hours; Fig. 2E), at least compared to β-actin (> 24 hours). In cells depleted of both of their GSK3 isoforms (GSK3α + GSK3β), Ras protein stability was markedly reduced by more than 3-fold, to reach a half-life of just ∼3.0 ± 1.6 hours (Fig. 2E). In cells transfected with the GSK3α or GSK3β siRNA, the half-life of Ras proteins was similar to that observed in the NT-transfected cells. Taken together, the results of Fig. 2 show that the two isoforms of GSK3 are regulating the stability of Ras family proteins in PC cells.

### LZTR1 is required for Ras protein degradation after GSK3 inhibition/depletion

**Figure 3:**
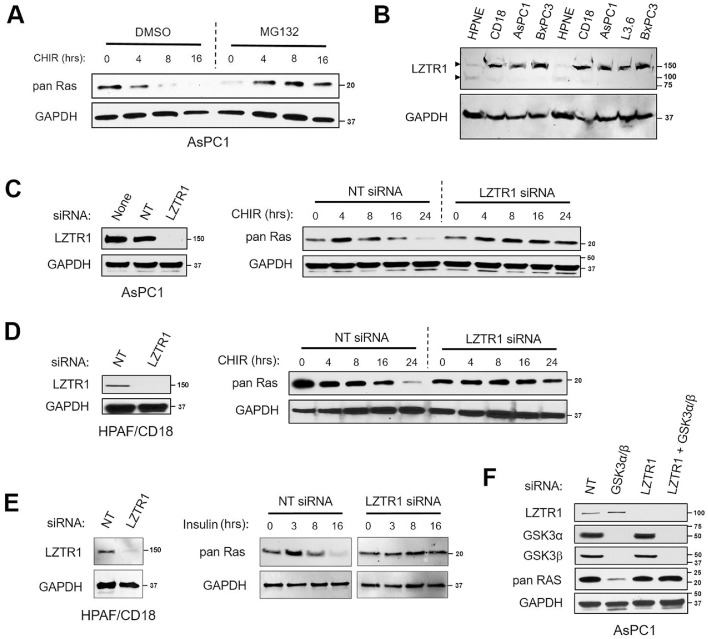
LZTR1 is required for the degradation of Ras proteins induced by the inhibition or silencing of GSK3. (A) Degradation of Ras proteins elicited by CHIR98014 is blocked by the proteasome inhibitor MG132. AsPC1 were first treated with proteasome inhibitor MG132 (20 µM) or else vehicle (DMSO). Two hours later, CHIR98014 (10 µM) was added and samples were collected at the indicated time points. (B) Levels of LZTR1 protein in a panel of four PC cell lines and HPNE cells. HPNE, HPAF/CD18, AsPC1, and BxPC3 cells were loaded in duplicates. Two arrows respectively point to the 150 kDa and 85 kDa species detected by the LZTR1 antibody. (C, D) LZTR1 is required for Ras protein degradation induced by CHIR98014. AsPC1 (C) and HPAF/CD18 (D) cells were transfected with LZTR1 siRNA or with a non-targeting siRNA (NT). Two days later, cells were exposed to CHIR98014 (10 µM), with samples collected at the indicated time points after CHIR98014. Left panels: LZTR1 levels two days post-transfection. (E) LZTR1 is required for Ras protein degradation induced by Insulin. HPAF/CD18 cells were transfected with LZTR1 siRNA or with a non-targeting siRNA (NT). Two days later, cells were exposed to Insulin Aspart (0.04 U/ml), with samples collected at the indicated time points after Insulin. Left panel: LZTR1 levels two days post-transfection. (F) LZTR1 is required for Ras protein degradation induced by the silencing of GSK3. AsPC1 cells were transfected with a non-targeting siRNA (NT) or with siRNA against LZTR1 (LZTR1) and/or the GSK3 kinases (GSK3α+β). Two days later, cells were analyzed for changes in Ras protein levels.

To assess the role of the ubiquitin-proteasome system (UPS) in the destabilization of Ras proteins induced by the inhibition of GSK3, we used proteasome inhibitor MG132. Prior to the addition of the GSK3 inhibitor, AsPC1 cells were pre-treated for 2 hours with MG132 (20 µM) or else vehicle (DMSO). In cells pre-treated with vehicle, CHIR98014 led to a sharp decline in Ras protein level (Fig. 3A). But in cells pre-treated with MG132, Ras proteins instead accumulated over time after the addition of CHIR98014. These results indicated that the UPS was involved in the loss of Ras proteins induced by the inhibition of GSK3.

The LZTR1 protein is a substrate receptor for E3 ubiquitin ligases that targets Ras proteins for proteasomal degradation, including Kras, Hras, Nras, and others [21,22,24]. LZTR1 uses its BTB-BACK domains to associate with Cul3 and its Kelch domain to interact with Ras proteins. In a panel of four PC cell lines, we detected LZTR1 as a 150 kDa protein (Fig. 3B). The protein was also detected in HPNE cells, a line of normal human pancreatic ductal cells immortalized with telomerase [41,42]. In HPNE cells, LZTR1 was expressed at much lower levels and was also detected as both a 150 kDa and 85 kDa protein (Fig. 3B).

To assess the role of LZTR1 in the regulation of Ras proteins by GSK3, we have silenced the expression of LZTR1 in AsPC1 and HPAF/CD18 cells. Two days after their transfection with an LZTR1 siRNA or non-targeting siRNA, cells were exposed to CHIR98014 and Ras proteins were monitored over time. In both the AsPC1 (Fig. 3C) and HPAF/CD18 (Fig. 3D) cells, knocking-down LZTR1 prevented the decline in Ras proteins induced by CHIR98014. This requirement for LZTR1 was also observed after the treatment of HPAF/CD18 cells with insulin (Fig. 3E). Insulin led to time-dependent decrease in the level of Ras proteins, but not in the LZTR1-depleted cells. The requirement for LZTR1 was also observed after the knockdown of the two GSK3 isoforms (Fig. 3F). AsPC1 cells were transfected with a non-targeting siRNA (NT) or with siRNAs against GSK3α+β only, LZTR1 only, or both LZTR1 and GSK3α+β. Two days later, cells were analyzed for differences in Ras proteins. As Fig. 3F shows, the silencing LZTR1 prevented the loss of Ras proteins induced by the knockdown of GSK3. Taken together, the results of Fig. 3 demonstrate that the LZTR1 protein is required for the degradation of Ras proteins induced by the silencing or inhibition of GSK3.

### GSK3 inhibition reduces PC cell proliferation, irrespective of KRAS mutations

In Fig. 3B, we detected high levels of LZTR1 protein in a panel of four PC cell lines. The panel included three lines carrying an oncogenic mutation in *KRAS* (AsPC1, HPAF/CD18, and L3.6pl) and the BxPC3 cells, known to be wild type for *KRAS* [49,50,55]. We examined the effects of GSK3 inhibition in each of these four cell lines, including its impact on Ras protein level, induction of apoptosis, and cell proliferation. HPNE cells, which also express the wild-type Kras protein, were included as a normal control.

**Figure 4:**
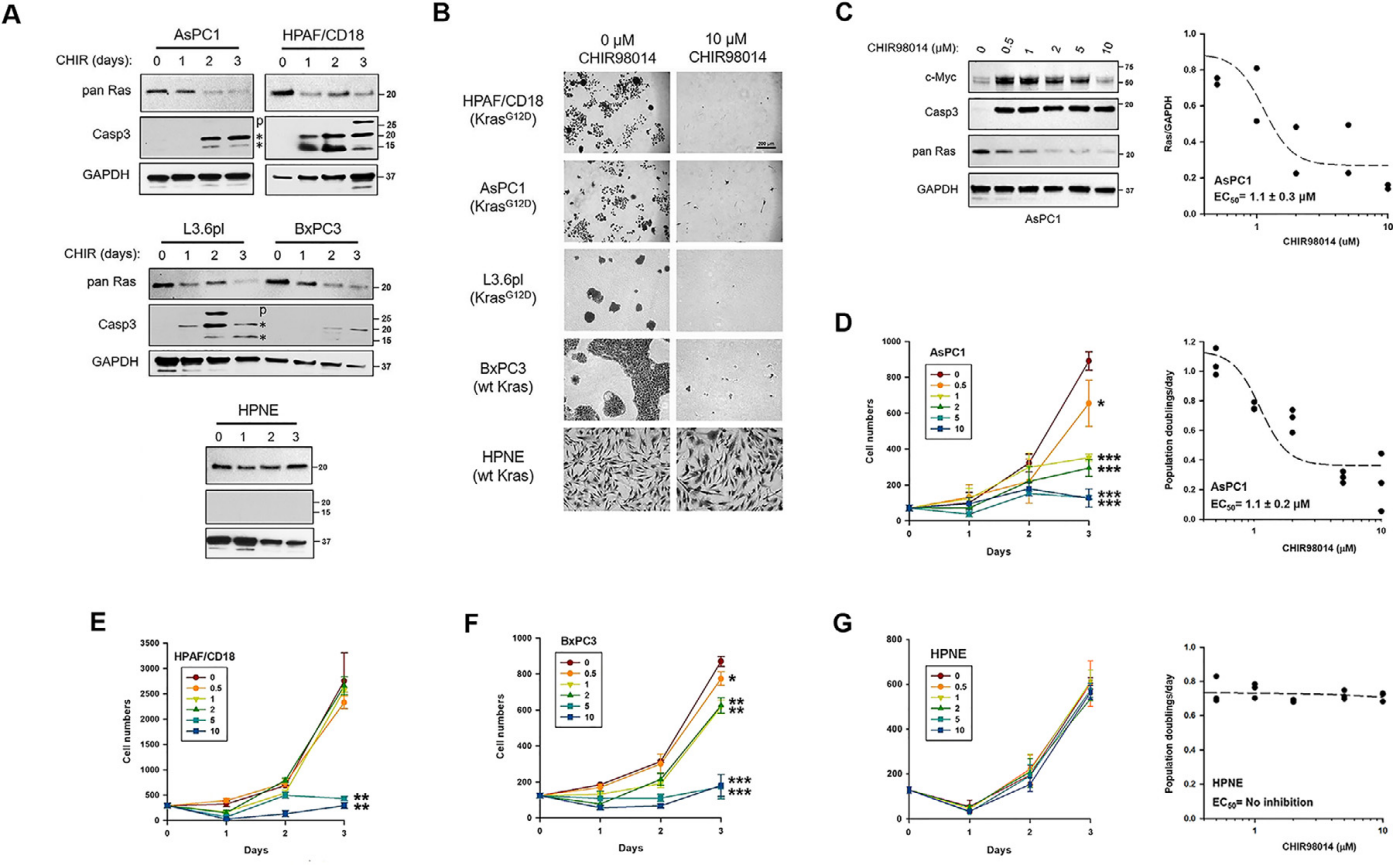
GSK3 inhibition in a panel of PC cell lines: its effects on Ras protein levels, cell proliferation, and apoptosis. The different cell lines were cultivated in the presence or absence of CHIR98014 to determine its effects on cell proliferation. Three of the PC lines carried an oncogenic *KRAS* mutation (AsPC1, HPAF/CD18, and L3.6pl) but the fourth line was wild type for *KRAS* (BxPC3). HPNE cells were included as a normal control. (A) GSK3 inhibition reduces Ras protein levels and induces apoptosis in mutant *KRAS*-expressing PC cells. The indicated cell lines were cultivated in the presence of 10 µM CHIR98014 for three days, with combined floating/adherent cells harvested on each day for Western blotting analysis of the levels of Ras proteins and cleaved caspase 3 (Casp 3), a markers of apoptosis. p, pro-caspase 3 precursor. * Cleaved caspase 3 fragments. (B) GSK3 inhibition blocks the proliferation of PC cells, irrespective of the mutational status of *KRAS*. Indicated cell lines were plated at low density and allowed to grow for three days in either the presence or absence of 10 µM CHIR98014, after which cells were stained with crystal violet. C) Dose-dependent effects of CHIR98014 on apoptosis and Ras protein levels. In duplicates, AsPC1 were exposed to the indicated concentrations of CHIR98014. Two days later, cells were analyzed for changes in Ras protein levels (pan Ras), markers of apoptosis (cleaved caspase 3) and markers of GSK3 kinase inhibition (c-Myc protein accumulation). Graph to the right shows the relative amount of Ras proteins detected (Ras/GAPDH) for each concentration of CHIR98014 (*n =* 2). Dotted line is a non-linear regression to a four parameter logistic curve. EC_50_ value ± SEM for the loss of Ras proteins by CHIR98014 is shown. D) GSK3 inhibition blocks the proliferation of AsPC1 cells. In triplicates, cells were cultivated in the presence of different concentrations of CHIR98014 (0, 0.5, 1, 2, 5, and 10 µM). Every day for three days, cells were set aside to be fixed, stained, and counted under the microscope. The number of cells per field is shown as a function of days in culture (mean ± SD; *n =* 3). Stars: Significantly different from the control samples (0 µM) in a Student's t-test at *p* < 0.05 (*), *p <* 0.01 (**), or *p <* 0.001 (***). Graph on the right shows the growth rate of the cells (in PD/day) for each concentration of CHIR98014 (*n =* 3). Dotted line is a non-linear regression to a four parameter logistic curve. EC_50_ value ± SEM for the inhibition of proliferation by CHIR98014 is shown. E-F) GSK3 inhibition block the proliferation of PC cells, irrespective of the mutational status of *KRAS*. In triplicates, HPAF/CD18 (E) and BxPC3 (F) cells were cultivated in the presence of different concentrations of CHIR98014 (0, 0.5, 1, 2, 5, and 10 µM), as described in panel D. The number of cells per field is shown as a function of days in culture (mean ± SD; *n =* 3). G) GSK3 inhibition fails to block proliferation of HPNE cells. In triplicates, HPNE cells were cultivated in the presence of different concentrations of CHIR98014, as in panel D. The number of cells per field is shown as a function of days in culture (mean ± SD; *n =* 3; left panel). Graph on the right shows the growth rate (in PD/day) for each concentration of CHIR98014 (*n =* 3). Dotted line is a linear least squares regression curve.

In a first experiment, the different cell lines were cultivated for 3 days in the presence of 10 µM CHIR98014. Once a day, cells were counted and set aside for Western blot analysis of Ras proteins and markers of apoptosis. At the end of the experiment, cells were crystal violet-stained and counted. In the four PC cell lines, CHIR98014 led to a time-dependent decline in the level of Ras proteins (Fig. 4A). HPNE cells express much less of the 150 kDa LZTR1 protein compared to PC cells and Ras proteins were not affected by the inhibitor. In the three PC cell lines that carried an oncogenic *KRAS* mutation, the decline in Ras proteins induced by CHIR98014 was accompanied by the induction of apoptosis, as shown by the induction of cleaved caspase 3 (Fig. 4A). In the BxPC3 cells, which only carry wild type *KRAS*, this apoptosis was only minimally induced after three days of treatment (Fig. 4A). In the HPNE cells, which express only a small amount of the 150 kDa LZTR1 protein, Ras proteins were not down-regulated by the drug and cleaved caspase 3 was also not induced. These results were reminiscent of those previously reported by Kazi et al. [40]. Similar to Kazi et al., GSK3 inhibition induced apoptosis in the mutant KRAS-expressing cell lines, but not in Ras-independent cell lines. Yet, when cells were counted at the end of the experiment, the growth of all four PC cell lines was equally and potently inhibited by the GSK3 inhibitor (Fig. 4B), irrespective of the mutational status of *KRAS* or induction of apoptosis.

To further investigate the relationship between the inhibition of GSK3, loss of Ras proteins, and induction of apoptosis, dose-response experiments were performed. In a first experiment, AsPC1 were exposed to different concentrations of CHIR98014 ranging from 0.5 µM to 10 µM. Two days later, cells were analyzed for differences in Ras protein levels (pan Ras) and markers of apoptosis (cleaved caspase 3) and of GSK3 kinase activity (cMyc accumulation). In cells treated with just 0.5 µM CHIR98014, the cMyc protein was already maximally up-regulated (Fig. 4C). The same was true for the induction of apoptosis. Yet, to reduce the level of Ras proteins, much higher doses of CHIR98014 were needed (Fig. 4C). The EC_50_ value for the loss of Ras proteins after CHIR98014 was determined to be equal to 1.1 ± 0.3 µM (Fig. 4C; graph on the right). These results indicated that the dose of GSK3 inhibitor needed to reduce Ras proteins was higher than that required to simply up-regulate cMyc and induce apoptosis.

In a second experiment, the different cells lines were cultivated in the presence of different concentrations of CHIR98014, also ranging from 0.5 µM to 10 µM. On four consecutive days, cells were counted once a day to determine growth rates (Fig. 4D-G). In AsPC1 cells, the drug inhibited proliferation in a dose-dependent manner (Fig. 4D). Proliferation was only minimally inhibited by the 0.5 µM dose, but was completely inhibited by the 5-10 µM concentrations. For each concentration of CHIR98014, we calculated the growth rate of the cells in population doublings per day (PD/day). In a dose-response curve, we plotted this growth rate as a function of CHIR98014 concentration (Fig. 4D; right panel). In AsPC1 cells, CHIR98014 led a dose-dependent decrease in proliferation with a calculated EC_50_ value of 1.1 ± 0.2 µM, in agreement with the concentrations needed to reduce Ras proteins (Right panels of Fig. 4C) as well as clonogenic growth (Fig. S1A). In HPAF/CD18 cells, proliferation was only minimally inhibited by CHIR98014 concentrations of 0.5 to 2 µM, but was completely blocked by the 5-10 µM doses (Fig. 4E). In HPAF/CD18 cells, the EC_50_ value for the inhibition of proliferation by CHIR98014 was 3.7 ± 0.3 µM (Fig. S3A). In the L3.6pl cells, the EC_50_ was calculated to be 2.4 ± 0.5 µM (Fig. S3B). In the Ras-independent BxPC3 cells expressing wild type Kras, proliferation was also inhibited by CHIR98014 in a dose-dependent manner (Fig. 4F). In BxPC3 cells, the EC_50_ value for the inhibition of proliferation was equal to 2.9 ± 0.5 µM (Fig. S3C). However, in the HPNE cells, which do not down-regulate Ras proteins in response to CHIR98014 (Fig. 4A), there were no inhibition of cell proliferation (Fig. 4G). These results show that in GSK3-inhibited cells, there is a direct relationship between the declining levels of Ras proteins and the inhibition of cell proliferation. They also show that these inhibitory effects are seen irrespective of the mutational status of *KRAS* or induction of apoptosis.

### Regulation of BxPC3 cell proliferation by the GSK3/LZTR1/Ras pathway

**Figure 5:**
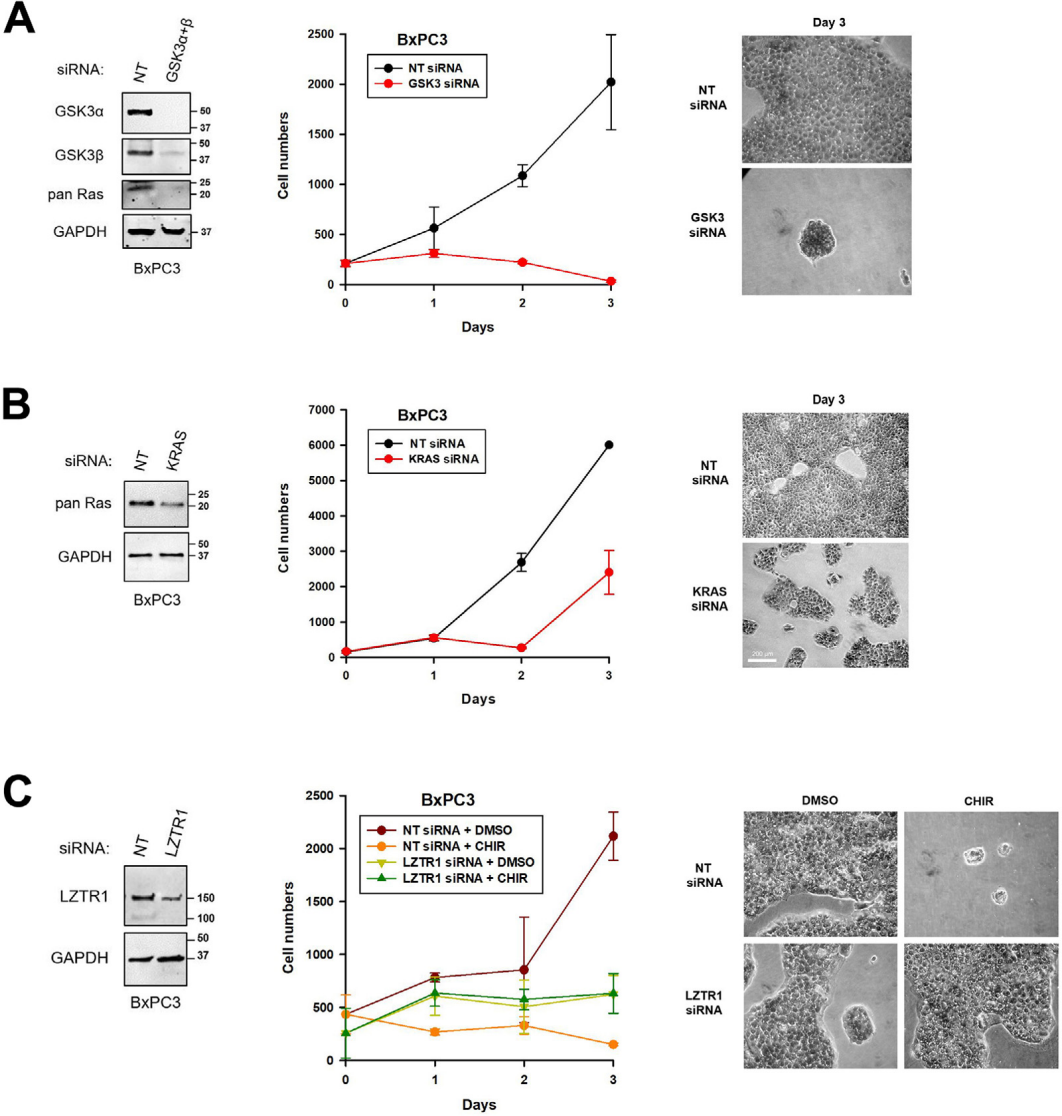
Regulation of PC cell proliferation by GSK3, KRAS, and LZTR1. (A) The knockdown of GSK3 inhibits the proliferation of BxPC3 cells. In triplicates, cells were transfected with a non-targeting siRNA (NT siRNA) or siRNA against both GSK3α and GSK3β (GSK3 siRNA). Starting the next day (Day 0), transfected cells were counted once a day for four days (days 0, 1, 2, and 3). To assess the knockdown, separate samples harvested two days after transfection (day 1) were analyzed by Western blotting (left panel). The middle panel shows the average number of cells counted per field as a function of days in culture (middle panel; mean ± SD; *n =* 3). On the last day, transfected cells were fixed and stained with crystal violet. Representative images of counted fields are shown (right panel). (B) The knockdown of KRAS inhibits the proliferation of BxPC3 cells. In triplicates, cells were transfected with a non-targeting siRNA (NT siRNA) or with siRNA against the KRAS mRNA (KRAS siRNA). Effects on cell proliferation (middle, and right panels) and assessment of the knockdown (left panel) were done as described in A. (C) The knockdown of LZTR1 eliminates the effects of CHIR98014 on cell proliferation. In triplicates, BxPC3 cells were transfected with a non-targeting siRNA (NT siRNA) or with siRNA against LZTR1 (LZTR1 siRNA). The next day, transfected cells were given fresh medium containing CHIR98014 (10 µM) or DMSO (vehicle). Cells were counted once a day for four days (days 0, 1, 2, and 3). The middle panel shows the average number of cells per field as a function of days in culture (middle panel; mean ± SD; *n =* 3). On the last day, transfected cells were fixed and stained with crystal violet. Representative images of counted fields are shown (right panel). To assess the LZTR1 knockdown, separate un-drugged samples were collected two days post-transfection (day 1) and analyzed by Western blotting (left panel).

Our results suggest the existence of a novel GSK3/LZTR1/Ras pathway that control the level of Ras proteins and proliferation of PC cells. To investigate the regulation of cell proliferation by this pathway, BxPC3 cells provide an ideal system to study proliferation without the confounding effects of ongoing apoptosis. We detect only minimal level of apoptosis in these cells after GSK3 inhibition (Fig. 4A). In a first experiment, BxPC3 cells were transfected with siRNA against both GSK3α and GSK3β (GSK3 siRNA) or else a non-targeting siRNA (NT siRNA). Plated cells were subsequently counted once a day for four days (on day 0, 1, 2, and 3). Two days post-transfection, un-drugged samples were collected separately to assess the knockdown. The analysis showed an almost complete knockdown of the two GSK3 isoforms and a reduced level of Ras proteins after transfection of the GSK3α+β siRNA (Fig. 5A; left panel). In the growth curves, cells transfected with the NT siRNA grew exponentially to reach high densities (Fig. 5A; middle and right panels). In contrast, those transfected with the GSK3α+β siRNA did not significantly increase in numbers. Identical results were also observed in HPAF/CD18 cells (Fig. S4A). Overall, these results show that, in PC cells, the knockdown of GSK3 is sufficient to inhibit cell proliferation.

To determine if the inhibition of proliferation could be recapitulated by the knockdown of KRAS alone, BxPC3 cells were transfected with a KRAS siRNA (KRAS siRNA) or with a non-targeting siRNA (NT siRNA). Western blot analysis performed 2 days post-transfection showed a reduced level of total Ras proteins after transfection of the KRAS siRNA (Fig. 5B; left panel). The reduction was of 57%, with the remaining Ras proteins likely represented by the Hras and Nras proteins, which the antibody also recognizes. In growth curves, BxPC3 cells transfected with the non-targeting siRNA grew much faster compared to those exposed to the *KRAS* siRNA (Fig. 5B; middle and right panels). Overall, these results show that reducing the level of wild type Kras protein is sufficient to inhibit the proliferation of BxPC3 cells. Identical results were also observed in HPAF/CD18 cells (Fig. S4B).

Next, we sought to determine if the effects of CHIR98014 on the proliferation of BxPC3 cells requires the LZTR1 protein. BxPC3 cells were transfected with an LZTR1 siRNA (LZTR1 siRNA) or non-targeting siRNA (NT siRNA). The next day, transfected cells were divided in two groups that were either cultivated in the presence of CHIR98014 (10 µM) or DMSO (vehicle). On four successive days, cells were counted (day 0, 1, 2, and 3). Western analysis performed two days post-transfection showed a 60% reduction in LZTR1 protein in cells transfected with the LZTR1 siRNA (Fig. 5C; left panel). In growth curves, cells transfected with the LZTR1 siRNA grew slower than those transfected with the NT siRNA (Fig. 5C; middle and right panels). However, cells transfected with the NT siRNA were sensitive to the GSK3 inhibitor and did not proliferate in the presence of 10 µM CHIR98014. In contrast, cells transfected by the LZTR1 siRNA initially grew to reach a plateau and their proliferation was not affected by CHIR98014 and grew equally fast in the presence or absence of the drug. These results show that the expression of LZTR1 is required for the inhibition of proliferation observed in BxPC3 cells after the inhibition of GSK3.

### GSK3 inhibitor reduces Ras proteins in implanted PC tumors and inhibits their growth

**Figure 6:**
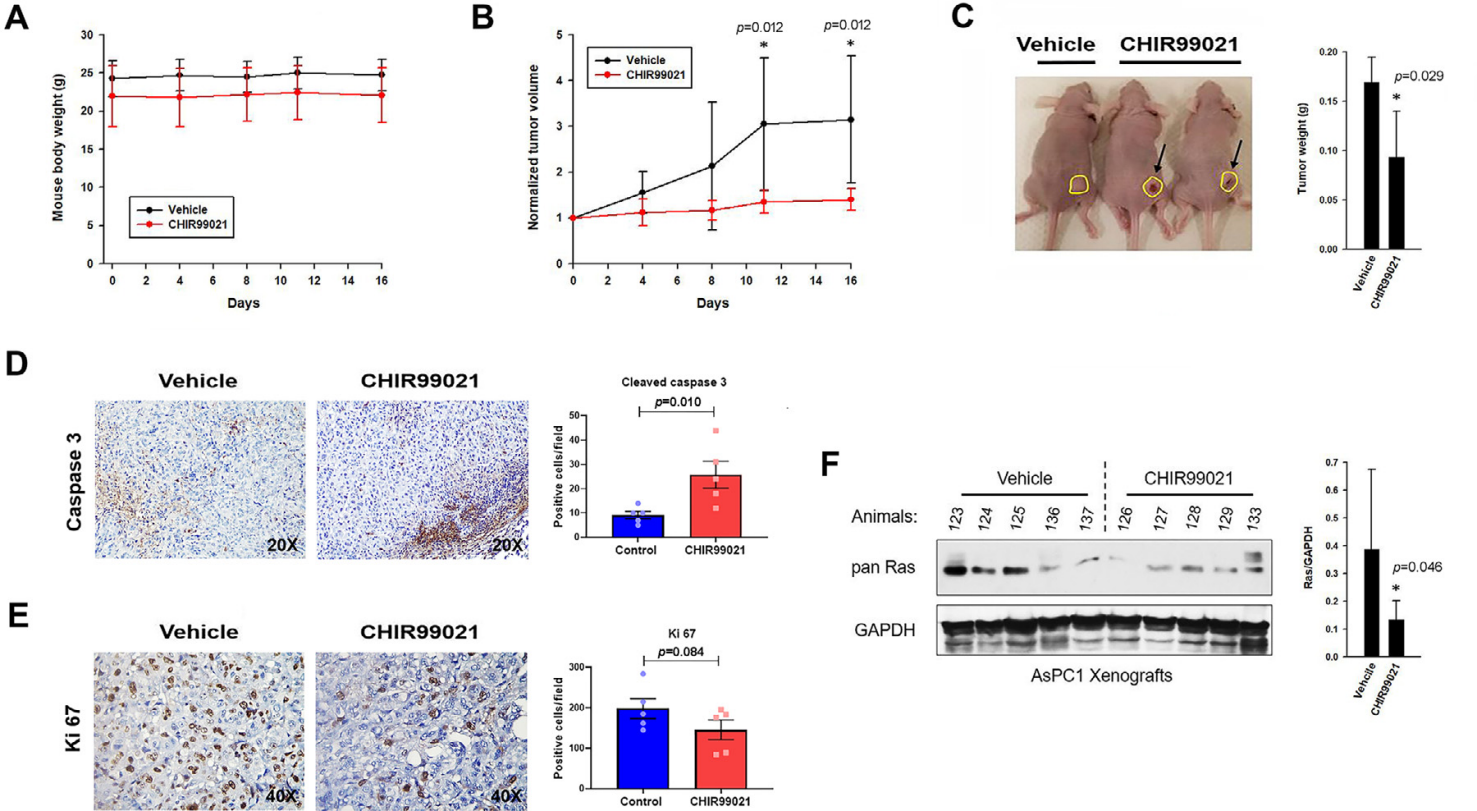
GSK3 inhibitor reduces Ras proteins in implanted PC tumors and inhibits their growth. AsPC1 cells were subcutaneously implanted in 10 athymic nude mice. Once animals developed palpable tumors, mice were randomized to two groups (*n =* 5 per group) receiving CHIR99021 (37.5 mg/kg twice/day by oral gavage) or else vehicle (PBS). After 16 days of treatment, mice were euthanized and tumors were harvested for analysis. A) CHIR99021 treatment did not affect body weights. Mice were weighted every 3-4 days, with their weights plotted as a function of days of treatment. B) CHIR99021 inhibited the growth of subcutaneously-implanted PC tumors. The volume of each tumor was measured with calipers every 3-4 days and expressed as a fold-change relative to their corresponding volume at day 0. * The difference between the two animals groups was statistically significant (*p =* 0.012, Mann-Whitney U test). C) CHIR99021 reduced weights of subcutaneously-implanted PC tumors. At the end of the experiment, tumors were harvested and weighted. In the CHIR99021-treated animals, dermal ulcerations were visible at the location of tumors (Arrows). Bar graph shows the difference in tumor weight between the groups reported as the mean ± S.D. (*n =* 5). * The difference between the two animals groups was statistically significant (*p =* 0.029, two-tailed Student's t test). D-E) IHC analysis of tumor specimens for markers of apoptosis and cell proliferation. Formalin-fixed paraffin-embedded tumors samples were stained with antibodies against cleaved caspase 3 and Ki-67. Representative light microscopic images of cytoplasmic staining for cleaved caspase 3 (D) and of nuclear staining for Ki-67 (E) are shown for each animal group. Graph to the right shows the number of positive cells per high power field, either for the individual tumors (dots) or as the mean ± SEM (bar graph). In tumors of CHIR99021-treated animals, the staining for cleaved caspase 3 was statistically higher than in the vehicle-treated group (*p =* 0.010, one-tailed Student's t test). A decrease in Ki-67 staining was also noted in tumors of CHIR99021-treated animals, albeit not to a statistically significant level (*p =* 0.084, one-tailed Student's t test). F) CHIR99021 reduces the level of Ras proteins in the implanted tumors. Harvested tumors were pulverized in liquid nitrogen, lysed in Laemmli buffer, and analyzed by Western blot for differences in levels of Ras family proteins. Bar graph shows the difference in Ras/GAPDH ratio between the groups reported as the mean ± S.D. (*n =* 5). *The difference between the two animals groups was statistically significant (*p =* 0.046, one-tailed Student's t test).

We tested the effects of a GSK3 inhibitor on the level of Ras proteins and the growth of implanted PC tumors. AsPC1 cells were subcutaneously implanted in ten athymic nude mice (10^6^ cells per site). Two weeks later, after animals had developed palpable tumors, mice were randomized into two groups (*n =* 5 per group) receiving half the maximum tolerated dose of CHIR99021 (37.5 mg/kg twice/day by oral gavage; [47]) or else vehicle. CHIR99021 is a CHIR98014 derivative with improved biodistribution and bioavailability [47]. Mice were treated 5 days/week for 16 days, after which the animals were sacrificed. CHIR99021 was well-tolerated and did not affect mouse body weights (Fig. 6A). Tumor volumes were measured with calipers twice a week (Fig. 6B). During the course of experiment, tumors grew in all of the vehicle-treated animals, albeit at very different rates for each tumor. In contrast, in the CHIR99021-treated animals, tumors did not substantially grow in any of the animals. At 11 and 16 days, these differences between the CHIR99021-treated and vehicle-treated groups were statistically significant (*p* = 0.012, Mann-Whitney U test). In the CHIR99021-treated animals, dermal ulcerations were also visible at the locations of the tumors (Fig. 6C; arrows). At the end of the experiment, tumors were harvested and weighted (Fig. 6C). Tumor weights were significantly reduced in the CHIR99021-treated group compared to the vehicle-treated animals (*p =* 0.029, Student's t test).

Harvested tumors were analyzed for differences in Ras protein levels, as well as markers of apoptosis and cell proliferation. At the time they were collected, tumors were split in halves, with one half saved for IHC analysis (Fig. 6D-E) and the other set aside for Western blot (Fig. 6F). IHC using the pan Ras antibody failed to label sufficient numbers of cells (≤ 1%) and could not be used to quantify Ras proteins. IHC analysis for markers of apoptosis and cell proliferation revealed a statistically significant increase in caspase 3 staining (Fig. 6D; *p =* 0.010) and a decrease in Ki-67 staining (Fig. 6E; *p =* 0.084) in the tumors of CHIR99021-treated animals compared to those of the vehicle-treated animals. As an alternative approach to quantify the levels of Ras proteins in tumor samples, Western blot analysis was performed on extracts of tumors. Levels of Ras proteins varied greatly among the vehicle-treated tumors, but levels were consistently lower in tumors of the CHIR99021-treated mice (Fig. 6F; *p =* 0.046). These results show that the level of Ras proteins and the growth of PC tumors can be reduced by the administration of a GSK3 inhibitor.

## Discussion

The LZTR1 protein uses its Kelch repeats (K1-K6) to interact with members of the Ras family, including the Kras, Hras, and Nras proteins [22,24,25]. LZTR1 can also associate with Cul3 to form an E3 ubiquitin ligase (BCR^LZTR1^) that poly-ubiquitinates Ras proteins and targets them for degradation [21,22,24]. But in spite of the potential importance of LZTR1 in Ras signaling, how these BCR^LZTR1^ complexes are regulated is still largely unknown. In this article, we show evidence that the function of LZTR1 is regulated by the GSK3 kinases, GSK3α and GSK3β. In a panel of four PC cell lines, levels of Ras proteins were markedly and consistently reduced after the inhibition (Figs. 1A, 3A, 3C-D, 4A, C) or the silencing of both GSK3 isoforms (Figs. 2A, 3F, 5A, S4A), as well as in PC cells treated with other structurally-unrelated GSK3 inhibitors, in particular SB216763 and lithium chloride (Fig. S5). The decline in Ras proteins was also observed under the physiological conditions of insulin stimulation (Figs. 1D, 3E), as well as in PC tumors of live animals treated with CHIR99021 (Fig. 6F). Follow-up studies indicated that this regulation of Ras protein level by GSK3 was mediated by changes in the stability of Ras proteins. Knocking-down both isoforms of GSK3 led to a three-fold decrease in the half-life of Ras family proteins (Fig. 2C-E). Importantly, both the 26S proteasome (Fig. 3A) and LZTR1 protein (Fig. 3C-F) were required for the degradation of Ras proteins induced by the inhibition/depletion of GSK3. In LZTR1-depleted PC cells, Ras protein level was no longer affected by the GSK3 inhibitor (Fig. 3C-D), insulin (Fig. 3E) or the knockdown of the GSK3 isoforms (Fig. 3F). Thus, the LZTR1 protein was determined to be critical for the loss of Ras proteins induced by the inhibition or silencing of GSK3.

In recent experiments, we investigated the regulation of LZTR1 function by GSK3. Inhibiting GSK3 did not cause LZTR1 level to increase in either the AsPC1 (Fig. S6A) or HPAF/CD18 cells (Fig. S6B). LZTR1 can physically interact with Ras proteins using its Kelch repeats [22,24,25]. To assess the impacts of GSK3 inhibition on the binding of Ras proteins to LZTR1, we have used a co-immunoprecipitation assay. AsPC1 cells were first treated with MG132 to block Ras protein degradation, after which cells were exposed or not to CHIR98014. Sixteen hours later, extracts were made and subjected to immunoprecipitation with an LZTR1 antibody. Ras proteins were captured by the LZTR1 antibody, but not by the normal mouse IgG control (Fig. S6C). Strikingly, an LZTR1/Ras interaction was detected, but only in those cells treated with CHIR98014. These results suggest a model according to which GSK3 inhibits LZTR1 in its ability to bind Ras proteins. LZTR1 uses Kelch repeats to interact with Ras proteins [22,24., 25., 26.] and several GSK3 consensus phosphorylation sites are located in and around these repeats (at T159, T266, T378, and S382). SDS-PAGE gels containing PhosTag^TM^ acrylamide can be used to separate proteins according to the extent of their phosphorylation [56]. In recent experiments, we used PhosTag^TM^ gels to analyze the phosphorylation of LZTR1 after the silencing of GSK3 (Fig. S6D). Unexpectedly, the silencing of GSK3 resulted in the hyper-phosphorylation of LZTR1. These results suggest the involvement of a second kinase, whose activity is directly or indirectly inhibited by GSK3. Follow-up studies will be needed to identify the phosphorylation events involved and the kinases and phosphatases responsible for their regulation. Consensus phosphorylation sites for GSK3 are also present in the Kras protein (at T35, T148), but changing these amino acids to alanine did not destabilize the Kras protein (data not shown).

One striking aspect of this regulation of Ras protein stability by GSK3 is its high requirement for the almost complete inhibition of GSK3. In the cycloheximide chase assays (Fig. 2E), knocking-down a single GSK3 isoform had little effects on Ras protein stability, whereas the silencing of both isoforms led to a 3-fold decrease in the half-life of these proteins. Also, for each of the three GSK3 inhibitors we have used (CHIR98014, SB216763, and lithium chloride), the concentrations needed to reduce Ras proteins were always higher than those typically required to inhibit the bulk of GSK3 activity (Figs. 4C, S5), as based on published EC_50_ values [51,57,58]. CHIR98014 reportedly stimulates the activity of glycogen synthase with an EC_50_ of 0.1 µM [51]. Likewise, when we treated AsPC1 cells with 0.5 µM CHIR98014, cMyc levels were already maximally induced (Fig. 4C), a telltale sign of GSK3 inhibition [59]. However, to reduce the level of Ras proteins, higher concentrations of inhibitor were clearly needed, up to 10 µM for complete Ras protein depletion (Fig. 4C). Are these higher requirements a reflection of off-target effects? We do not think so, given that the effects on Ras protein levels were also observed after the silencing of GSK3 (Figs. 2,3F) and exposure of the cells to insulin (Figs. 1D, 3E), which we know inhibits GSK3 under physiological conditions. Instead, we are proposing that the requirements for the higher doses of inhibitors are a reflection of the biochemical properties of GSK3 itself or alternatively, of the specific GSK3 substrate involved. GSK3 is often found in large complexes where it interacts with other proteins, some of which carrying GSK3β-interacting domains 60., 61., 62.. In some cases, as in the β-catenin destruction complex, the kinase itself is an integral part of the complex. If GSK3 is part of a complex that regulates LZTR1, the kinase could control the function of this complex in more than one way, using both protein-protein interactions and its own kinase activity to block the activation of BCR^LZTR1^ complexes. Activating LZTR1 could thus require, not only the inhibition of GSK3’s kinase activity, but also the disruption of these protein-protein interactions. For disrupting these interactions, the pharmacological inhibition of the kinase's active center with a drug, such as CHIR99014, may not be as efficacious as the knockdown of GSK3 or its S9/S21-phosphorylation, which causes vast conformational changes [63,64]. Another possibility could be that the GSK3 substrate involved contains multiple GSK3 phosphorylation sites, which are acting together to control the biochemical activity of the protein. In regulatory proteins, multisite phosphorylation can produce switch-like transitions but at higher thresholds and EC_50_ values 65., 66., 67., 68.. The LZTR1 protein itself contains many putative GSK3 phosphorylation sites in and around its Kelch domains (at T159, T266, T378, and S382) and would be a good candidate for this type of regulation, if GSK3 happens to be directly involved.

Two types of biological responses to GSK3 inhibition/depletion were observed in cultivated PC cells. The first type of response was apoptosis, which we detected using cleaved caspase 3 as a marker. Concomitantly with the loss of Ras proteins, apoptosis was induced in the three PC cell lines that carried an oncogenic KRAS mutation, but only minimally in the BxPC3 cells expressing wild type Kras. This selectivity for the killing of cancer cell potentially addicted to oncogenic KRAS is reminiscent of the apoptotic response to GSK3 inhibition previously described by Kazi et al. [40]. As in Kazi et al., the apoptosis was induced as soon as the bulk of GSK3 was inhibited, such as when AsPC1 cells were exposed to 0.5 µM CHIR98014 (Fig. 4C). At this concentration, cMyc was already maximally induced, presumably because of its reduced T58 phosphorylation by GSK3. In Kazi et al., this accumulation of cMyc was necessary and sufficient for the induction of apoptosis after GSK3 inhibition [40]. But in spite of its potential value for the selective killing of Ras-addicted cancer cells, this apoptotic response to GSK3 inhibition did not appear to have a strong impact on the overall proliferation of the cells. When cultivated in the presence of 0.5 µM CHIR98014, AsPC1 grew almost as fast as the untreated controls (1.05 ± 0.09 PD/day versus 1.21 ± 0.03 PD/day; *n =* 3) (Fig. 4D), in spite of the already maximal induction of apoptosis by the drug (Fig. 4C). We thus concluded that the apoptotic response to GSK3 inhibition must therefore only affect a small fraction of the cells, not enough to impede population growth.

The second type of response to GSK3 deficiency was a reduction in cell proliferation and clonogenic growth, which we have observed at the higher concentrations of CHIR98014 (1-5 µM range). In AsPC1 cells, the inhibition of proliferation (EC_50_=1.1 ± 0.2 µM; Fig 4D) and clonogenic growth (EC_50_=1.7 ± 0.2 µM; Fig. S1A) by CHIR98014 correlated with the declining levels of Ras proteins (EC_50_=1.1 ± 0.3 µM; Fig. 4C). This inhibition of proliferation was seen in all four PC cell lines, irrespective of the mutational status of *KRAS* or induction of apoptosis. In both wild type and mutant *KRAS*-expressing PC cells, a reduction in cell proliferation was also observed after the knockdown of the two GSK3 isoforms (Figs. 5A, S4A) or KRAS itself (Figs. 5B, S4B). Moreover, in BxPC3 cells transfected with LZTR1 siRNA, the inhibition of proliferation by CHIR98014 was no longer observed and the cells grew as fast with or without 10 µM CHIR98014 (Fig. 5C). These results show that the loss of proliferation observed in GSK3-inhibited PC cells is a direct consequence of LZTR1 function and its impacts on the level of Ras proteins.

GSK3 plays an important role in PC development 33., 34., 35., 36. and GSK3 inhibitors have shown promises in animal models of PC and other malignancies [35,38,39,69]. Several clinical trials of GSK3 inhibitor 9‑ING‑41 are now underway in patients with advanced solid tumors (NCT03678883, NCT04239092, NCT05010629, and NCT04832438). In Kazi et al. [40], the GSK3 kinases were reported to be essential to the viability of Ras-addicted cancer cells, but dispensable to Ras-independent cancer cells [40]. This selective targeting of Ras-addicted cells makes the GSK3 kinases ideal targets for the treatment of Ras-driven malignancies, such as PC. However, as suggested by our results, this apoptosis appears to affect only a small fraction of Ras-addicted PC cells, not enough to impact population growth. Unless, new approaches are developed to maximize this apoptotic response so as to affect the bulk of the tumor cells, the drugs are unlikely to have an impact on patient survival. Instead, dose-limiting toxicities driven by the activation of LZTR1 could have an impact on normal tissues. The LZTR1 protein is expressed in many normal tissues, based on GEO profiles, and the Ras proteins are critical regulators of many normal processes. Because LZTR1 interacts with many members of the Ras family [21,22,24], Ras signaling in normal tissues could be affected by a GSK3 inhibitor. On the other hand, in our mouse studies, the GSK3 inhibitor did not appear to have any obvious side effects (Fig. 6A), except for the inhibition of PC tumor growth (Fig. 6B-E). Future and ongoing human clinical trials will be needed to determine if the benefit of these inhibitors for cancer therapy outweighs their potential risks of toxicity to normal tissues.

## CRediT authorship contribution statement

**Chitra Palanivel:** Methodology, Validation, Investigation, Visualization. **Neha Chaudhary:** Validation, Investigation. **Parthasarathy Seshacharyulu:** Investigation. **Jesse L. Cox:** Formal analysis, Data curation. **Ying Yan:** Investigation. **Surinder K. Batra:** Writing – review & editing, Supervision, Project administration, Funding acquisition. **Michel M. Ouellette:** Conceptualization, Validation, Formal analysis, Data curation, Writing – original draft, Writing – review & editing, Visualization, Supervision, Project administration, Funding acquisition.

## Declaration of Competing Interest

The authors declare that they have no conflict of interest.

## Appendix Supplementary materials

Image, application 1
