# Supplementary material for: The GSK3 kinase and LZTR1 protein regulate the stability of Ras family proteins and the proliferation of pancreatic cancer cells
Source: Neoplasia. 2022 Jan 31;25:28–40. doi: 10.1016/j.neo.2022.01.002 (PMC8814762; doi:10.1016/j.neo.2022.01.002)
Supplement: Supplementary file 1 [file mmc1.pdf]

SUPPLEMENTAL DATA

MATERIALS AND METHODS

Materials

SB216763 was purchased from Selleck Chemicals (Houston, TX). Lithium chloride was from Sigma-Aldrich (Saint-Louis, MO). Protein A/G-agarose beads were from Santa Cruz Biotech (Dallas, TX). Other reagents are described in the Materials and Methods of the main article.

Clonogenic Assays

In 6-well plates, cells were seeded in duplicates at 2000 cells/well. The next day, cells were feed fresh medium containing different concentrations of CHIR98014 (0 to 10 μM). After 24 hours of cultivation, the drug was removed and the cells were washed once and then given fresh medium. After 2 weeks of incubation, colonies were stained with crystal violet and counted with the help of the ImageJ program. Colony numbers were plotted as a function of the concentration of CHIR98014 to produce a dose-response curve. For each curve, an EC_{50} value was calculated by non-linear regression and fitted to a four parameter logistic curve by SigmaPlot v. 11.

Co-immunoprecipitations

AsPC1 cells were first exposed to MG-132 (20 μM) to block Ras protein degradation. Two hours later, CHIR98014 (10 μM) or vehicle (DMSO) were added to the treated cells. Sixteen hours after CHIR98014/DMSO, cells were washed twice with PBS and lysed in ice-cold IP buffer (25 mM Tris-HCl, 150 mM NaCl, 1 mM EDTA, 0.5% NP-40, pH 7.4). Cell debris were pelleted by centrifugation (10,000 x g for 10 min. at 4°C) and supernatants were either subjected to immunoprecipitation or stored at -80°C. Co-IPs were performed according to the manufacturer's instructions (Santa Cruz Biotechnology, Inc.). Briefly, an equal volume of each extract (400-500

μl; 400-500 μg protein) was thawed on ice, after which 2 μg of LZTR1 antibody (cat# sc390166; Santa Cruz Biotechnology) or normal mouse IgG (cat# sc-2025; Santa Cruz Biotechnology) were added. After 4 hours of rotation at 4°C, we added 20 μl of pre-washed protein A/G-agarose beads to each sample (50% v/v; Santa Cruz Biotech, Dallas, TX). After another 6-16 hours of rotation at 4°C, beads were recovered by centrifugation (2,500 rpm for 5 min) and washed 4 times with 1 ml each of ice-cold IP buffer. Beads were resuspended in Laemmli buffer and the eluted proteins were analyzed by Western blot for the presence of Ras proteins, using both the pan Ras and Ras^{G12D} antibodies.

Electrophoretic analysis of LZTR1 on PhosTag™ acrylamide gels.

AsPC1 cells were transfected with a non-targeting siRNA (NT) or siRNA against GSK3α, GSK3β, or both kinases. Two days later, samples were harvested in Laemmli buffer and an equal volume of each sample were resolved by electrophoresis on a 4% SDS-PAGE gel containing 25 μM of PhosTag™ acrylamide (APExBIO, Boston, MA) and 50 μM $MnCl_2$. Made according to the manufacturer's instructions, the gel was run at 30 mA/gel until the bromophenol blue dye reached the bottom. After electrophoresis, the gel was treated with 10 mM EDTA to facilitate Western blot transfer. Membrane was subsequently probed with the anti-LZTR1 antibody.

SUPPLEMENTARY FIGURE LEGENDS

Figure S1. GSK3 inhibition reduces colony formation by AsPC1 and HPAF/CD18 cells. In duplicates, AsPC1 **(A)** and HPAF/CD18 **(B)** cells were plated at clonal densities (2000 cells/35 mm dish). The next day, cells were exposed to different concentrations of CHIR98014 for 24 hours, after which cells were feed fresh medium without drug. Two weeks later, colonies were stained and counted. Right panels show the relative number of colonies (compared to the no-drug samples) for each concentrations of CHIR98014 (n=2). Dotted line is a non-linear regression to a

four parameter logistic curve. EC_{50} value ± SEM for the inhibition of colony formation by CHIR98014 is shown for both cell lines.

Figure S2. GSK3 inhibition reduces Ras signaling and the level of Ras proteins in HPAF/CD18 cells. HPAF/CD18 cells were harvested at the indicated time points after the addition of CHIR98014 (10 µM). Samples were Western blotted with antibodies against the indicated proteins, including Ras family proteins (pan Ras) and their G12D mutant proteins (Ras^{G12D}). Phosphorylated and total cMyc proteins were used as surrogate markers of GSK3 kinase activity. The experiment was performed twice with same outcome.

Figure S3. GSK3 inhibition block the proliferation of HPAF/CD18 and BxPC3 cells. In triplicates, cells were cultivated in the presence of different concentrations of CHIR98014 (0, 0.5, 1, 2, 5, and 10 µM). Every day for three days, cells were counted under the microscope. The panels show the growth rate of HPAF/CD18 **(A)**, L3.6pl **(B)**, and BxPC3 **(C)** cells for each concentrations of CHIR98014 (in PD/day; n=3). Dotted line is a non-linear regression to a four parameter logistic curve. EC_{50} values ± SEM for the inhibition of proliferation by CHIR98014 are shown for both cell lines.

Figure S4. Regulation of HPAF/CD18 cell proliferation by GSK3 and KRAS. (A) The knockdown of GSK3 inhibits the proliferation of HPAF/CD18 cells. In triplicates, cells were transfected with a non-targeting siRNA (NT siRNA) or siRNA against both GSK3α and GSK3β (GSK3 siRNA). Starting the next day (Day 0), transfected cells were counted once a day for four days (days 0, 1, 2, and 3). To assess the knockdown, separate samples harvested two days after transfection (day 1) were analyzed by Western blotting (left panel). The middle panel shows the average number of cells counted per field as a function of days in culture (middle panel; mean ±

SD; n=3). On the last day, transfected cells were fixed and stained with crystal violet. Representative images of counted fields are shown (right panel). **(B)** The knockdown of KRAS inhibits the proliferation of HPAF/CD18 cells. In triplicates, cells were transfected with a non-targeting siRNA (NT siRNA) or with siRNA against the KRAS mRNA (KRAS siRNA). Effects on cell proliferation (middle, and right panels) and assessment of the knockdown (left panel) were done as described in A.

Figure S5: Dose-dependent effects of other GSK3 inhibitors on levels of Ras proteins. AsPC1 were exposed to the indicated concentrations of SB216763 **(A)** or lithium chloride **(B)**. After 24 hours, cells were analyzed by Western blotting for changes in Ras proteins, using both the pan Ras and Ras^{G12D} antibodies.

Figure S6. GSK3 regulates the association of LZTR1 with Ras family proteins. (A-B) Levels of LZTR1 protein are not induced by the inhibition of GSK3. AsPC1 (A) and HPAF/CD18 (B) cells were harvested at the indicated time points after the addition of CHIR98014 (10 μM). **(C)** GSK3 inhibition induces the binding of Ras proteins to LZTR1. AsPC1 cells were first exposed to MG132 (20 μM) in order to prevent Ras protein degradation. Cells were then treated for 16 hours with either CHIR98014 (10 μM) or else vehicle (DMSO). Cells were lysed and the extracts were subjected to immunoprecipitation with either an LZTR1 antibody (αLZTR1) or normal mouse IgG control as a control (IgG). Extracts (input) and immunoprecipitated proteins (IP) were analyzed for the presence of Ras proteins (pan Ras, Ras^{G12D}). (D) Electrophoretic analysis of the LZTR1 protein on PhosTag™ gels. AsPC1 cells were transfected with a non-targeting siRNA (NT) or with siRNA against GSK3α, GSK3β, or both kinases. Two days later, samples were harvested in Laemmli buffer and an equal volume of each sample were resolved by electrophoresis on a 4% SDS-PAGE gel containing PhosTag™ acrylamide. Following electrophoresis, the gel was treated with 10 mM EDTA and proteins were transferred to a membrane, which was then probed with the

LZTR1 antibody. In the NT-transfected cells, LZTR1 was detected as both a slow migrating band (arrow a) and a fast migrating form (arrow b), which respectively correspond to hyper- and hypo-phosphorylated forms of the protein. Both short (SE) and long (LE) exposures of the membrane are shown for the NT-transfected cells.

Figure S1

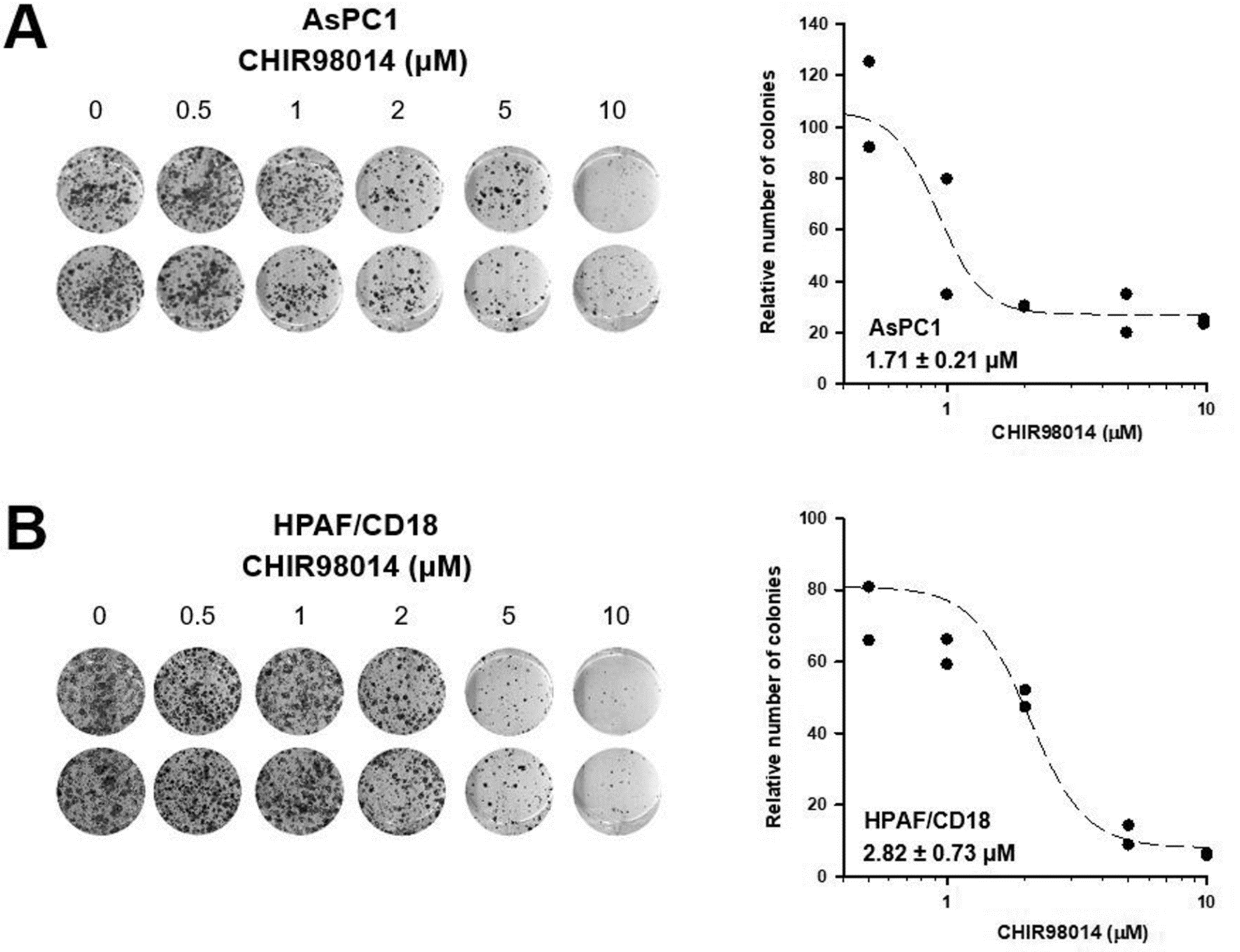
A
AsPC1
CHIR98014 (µM)
0
0.5
1
2
5
10
Relative number of colonies
140
120
100
80
60
40
20
0
AsPC1
1.71 ± 0.21 µM
1
10
CHIR98014 (µM)
B
HPAF/CD18
CHIR98014 (µM)
0
0.5
1
2
5
10
Relative number of colonies
100
80
60
40
20
0
HPAF/CD18
2.82 ± 0.73 µM
1
10
CHIR98014 (µM)

Figure S2

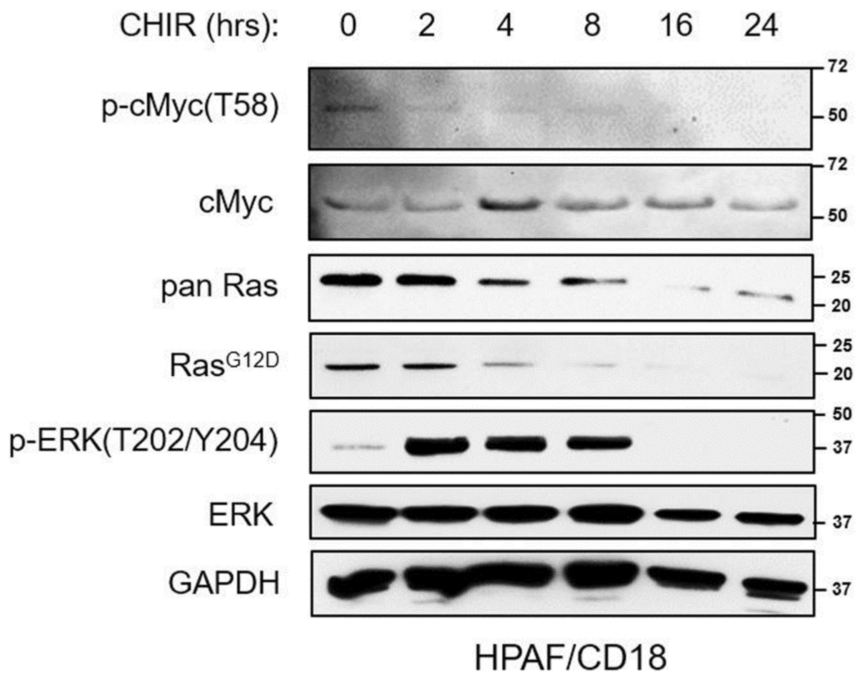
CHIR (hrs): 0 2 4 8 16 24
p-cMyc(T58)
cMyc
pan Ras
Ras^{G12D}
p-ERK(T202/Y204)
ERK
GAPDH
72
50
72
50
25
20
25
20
50
37
37
37
HPAF/CD18

Figure S3

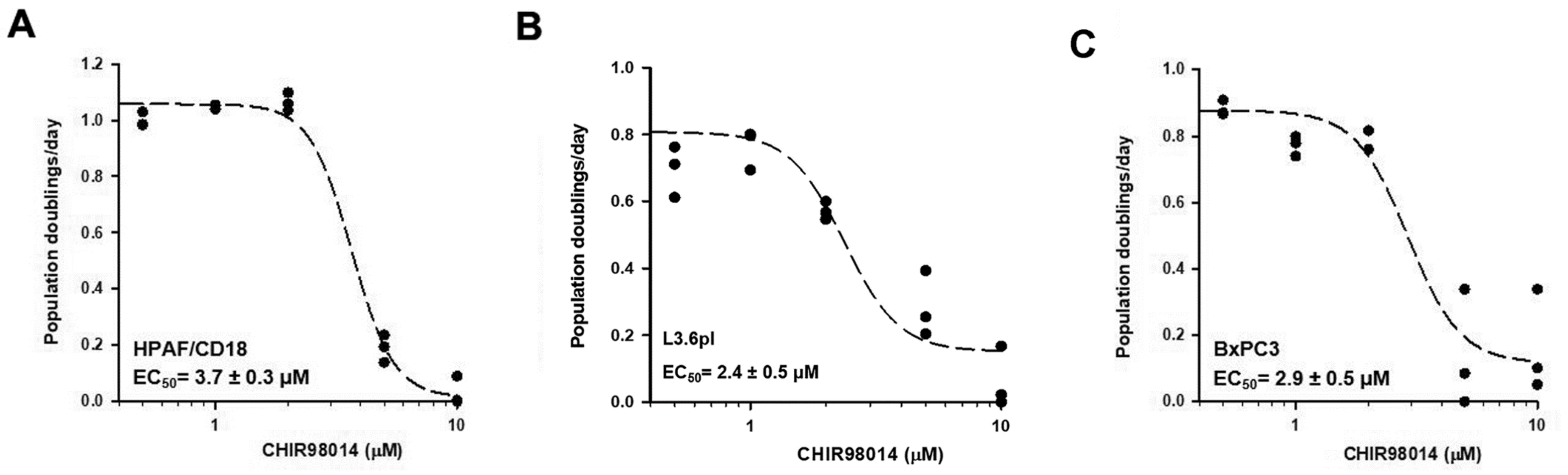
A
Population doublings/day
1.2
1.0
0.8
0.6
0.4
0.2
0.0
HPAF/CD18
EC_{50}= 3.7 ± 0.3 μM
1
10
CHIR98014 (μM)
B
Population doublings/day
1.0
0.8
0.6
0.4
0.2
0.0
L3.6pl
EC_{50}= 2.4 ± 0.5 μM
1
10
CHIR98014 (μM)
C
Population doublings/day
1.0
0.8
0.6
0.4
0.2
0.0
BxPC3
EC_{50}= 2.9 ± 0.5 μM
1
10
CHIR98014 (μM)

Figure S4

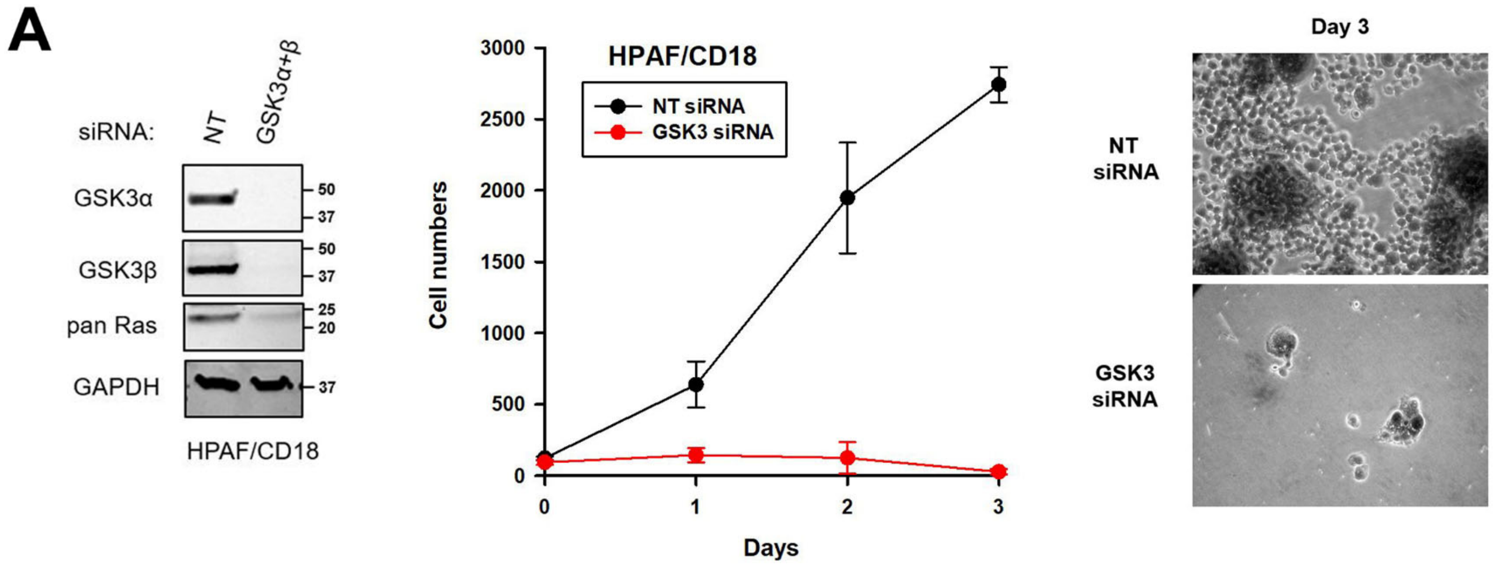
A
siRNA:
NT
GSK3α+β
GSK3α
GSK3β
pan Ras
GAPDH
50
37
25
20
HPAF/CD18
HPAF/CD18
NT siRNA
GSK3 siRNA
Cell numbers
Days
Day 3
NT siRNA
GSK3 siRNA

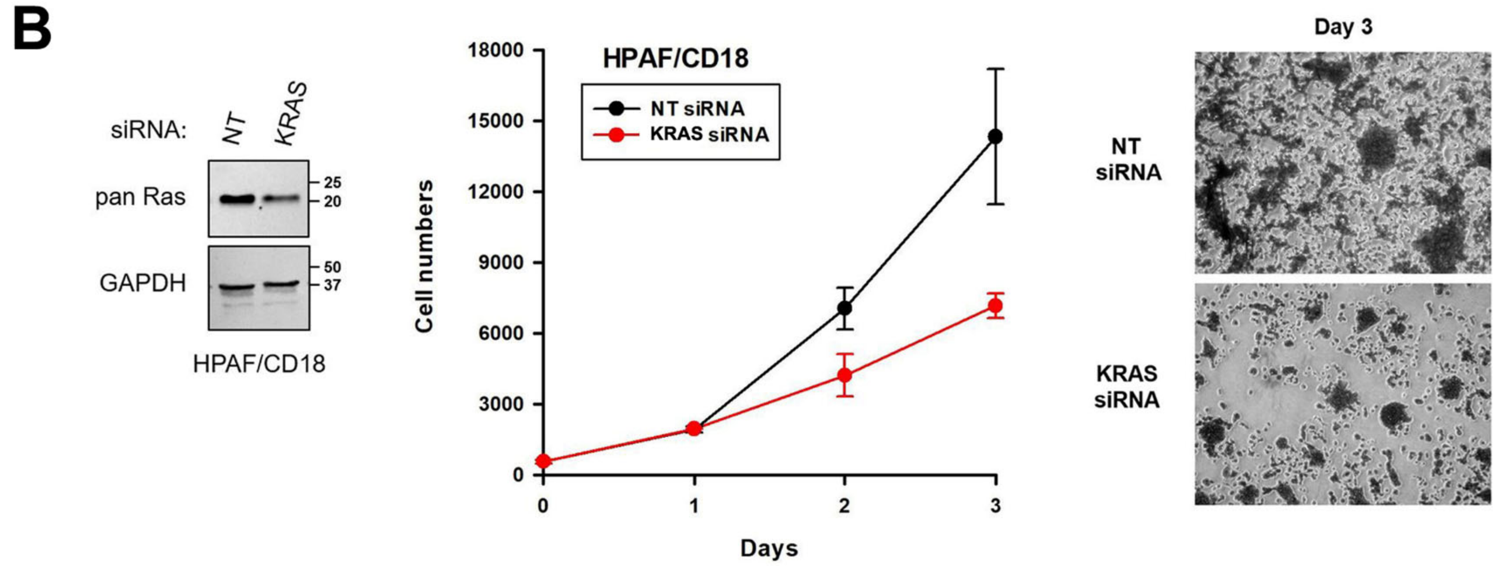
B
siRNA:
NT
KRAS
pan Ras
GAPDH
25
20
50
37
HPAF/CD18
HPAF/CD18
NT siRNA
KRAS siRNA
Cell numbers
Days
Day 3
NT siRNA
KRAS siRNA

Figure S5

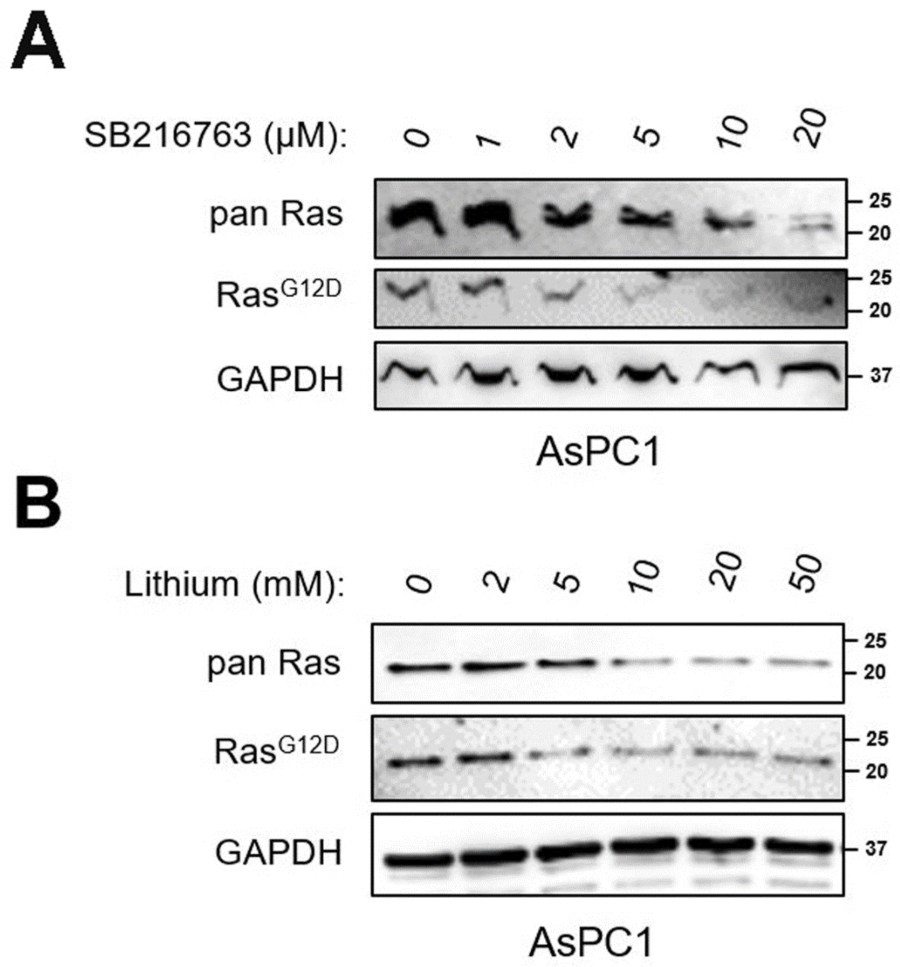
A
SB216763 (μM):
0
1
2
5
10
20
pan Ras
RasG12D
GAPDH
25
20
25
20
37
AsPC1
B
Lithium (mM):
0
2
5
10
20
50
pan Ras
RasG12D
GAPDH
25
20
25
20
37
AsPC1

Figure S6

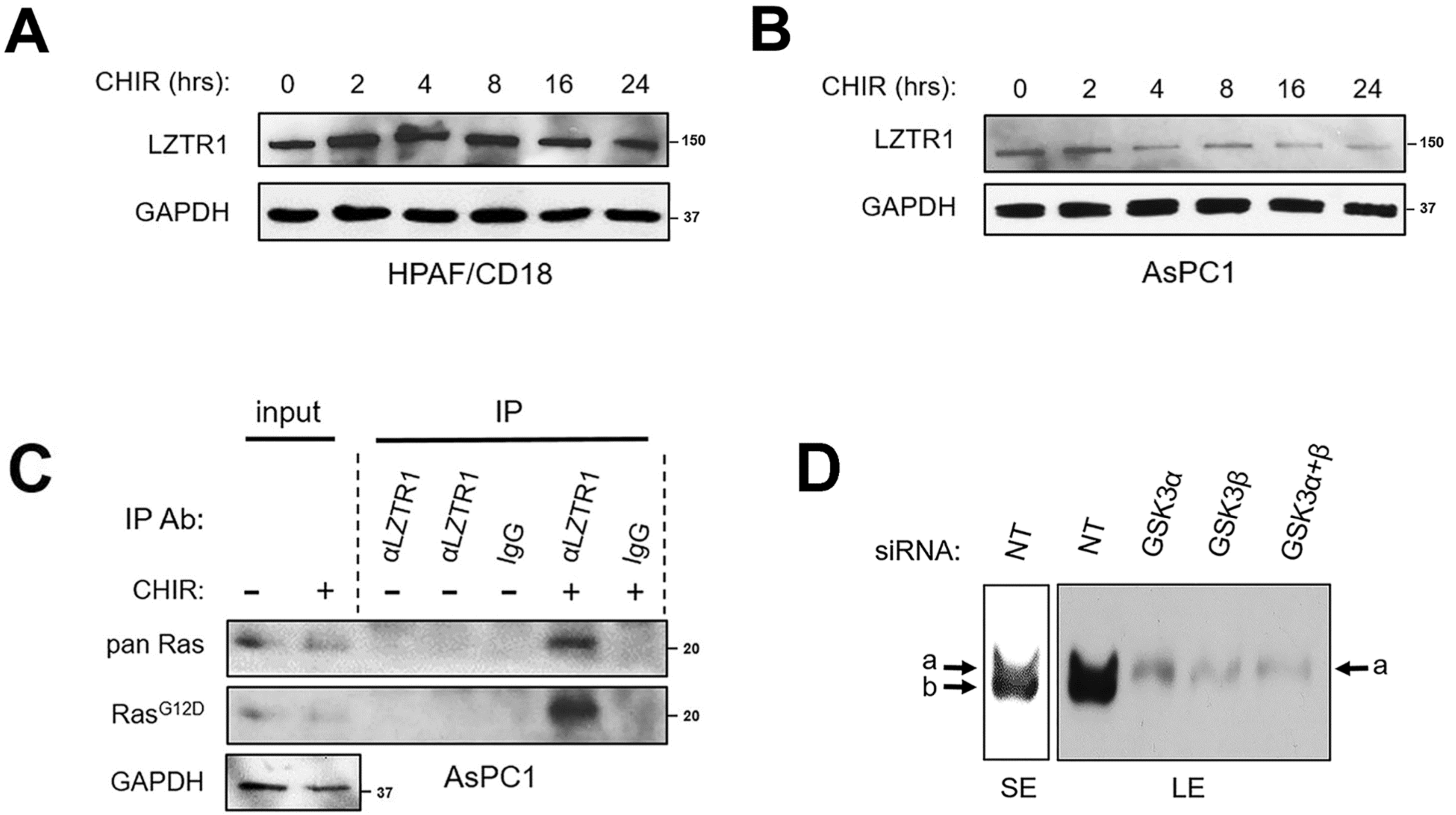
A
CHIR (hrs): 0 2 4 8 16 24
LZTR1
GAPDH
150
37
HPAF/CD18
B
CHIR (hrs): 0 2 4 8 16 24
LZTR1
GAPDH
150
37
AsPC1
C
input
IP
IP Ab:
αLZTR1
αLZTR1
IgG
αLZTR1
IgG
CHIR:
– + – – – + +
pan Ras
Ras^{G12D}
GAPDH
20
20
37
AsPC1
D
siRNA:
NT
NT
GSK3α
GSK3β
GSK3α+β
a
b
a
SE
LE
